# Transcriptome deregulation of peripheral monocytes and whole blood in *GBA*-related Parkinson’s disease

**DOI:** 10.1186/s13024-022-00554-8

**Published:** 2022-08-17

**Authors:** Giulietta Maria Riboldi, Ricardo A. Vialle, Elisa Navarro, Evan Udine, Katia de Paiva Lopes, Jack Humphrey, Amanda Allan, Madison Parks, Brooklyn Henderson, Kelly Astudillo, Charalambos Argyrou, Maojuan Zhuang, Tamjeed Sikder, J. Oriol Narcis, Shilpa Dilip Kumar, William Janssen, Allison Sowa, Giacomo P. Comi, Alessio Di Fonzo, John F. Crary, Steven J. Frucht, Towfique Raj

**Affiliations:** 1 The Marlene and Paolo Fresco Institute for Parkinson’s Disease and Movement Disorders, New York University Langone Health, 222 East 41st street, New York, NY 10017 USA; 2 Nash Family Department of Neuroscience & Friedman Brain Institute, Icahn School of Medicine at Mount Sinai, One Gustave L. Levy Place, New York, NY 10029 USA; 3 Ronald M. Loeb Center for Alzheimer’s disease, Icahn School of Medicine at Mount Sinai, One Gustave L. Levy Place, New York, NY 10029 USA; 4 Department of Genetics and Genomic Sciences & Icahn Institute for Data Science and Genomic Technology, Icahn School of Medicine at Mount Sinai, One Gustave L. Levy Place, Box 1498, New York, NY 10029 USA; 5 Estelle and Daniel Maggin Department of Neurology, Icahn School of Medicine at Mount Sinai, One Gustave L. Levy Place, Box 1137, New York, NY 10029 USA; 6 Rush Alzheimer’s Disease Center, Rush University Medical Center, Chicago, IL USA; 7 Department of Biochemistry and Molecular Biology (Universidad Complutense de Madrid) & Centro de Investigación Biomédica en Red sobre Enfermedades Neurodegenerativas (CIBERNED), Madrid, Spain; 8 Department of Pathology, Icahn School of Medicine at Mount Sinai, 1468 Madison Avenue, Annenberg Building, 15th Floor, New York, NY 10029 USA; 9 Neuropathology Brain Bank & Research CoRE, Icahn School of Medicine at Mount Sinai, 1425 Madison Avenue, Room 9-22, New York, NY 10029 USA; 10 Microscopy Core and Advanced Bioimaging Center at the Icahn School of Medicine at Mount Sinai Center, 1468 Madison Avenue, Room 18-250, New York, NY 10029 USA; 11 Fondazione IRCCS Ca’ Granda Ospedale Maggiore Policlinico, Neurology Unit, Milan, Italy; 12 Dino Ferrari Center, Neuroscience Section, Department of Pathophysiology and Transplantation, University of Milan, Via Francesco Sforza, 35, 20122 Milano, MI Italy; 13 Icahn School of Medicine at Mount Sinai, 1425 Madison Avenue, ICAHN 10-70E, New York, NY 10029–6574 USA

**Keywords:** Parkinson’s disease, Monocytes, *GBA*, beta-glucocerebrosidase, Transcriptomic analysis

## Abstract

**Background:**

Genetic mutations in beta-glucocerebrosidase (*GBA*) represent the major genetic risk factor for Parkinson’s disease (PD). *GBA* participates in both the endo-lysosomal pathway and the immune response, two important mechanisms involved in the pathogenesis of PD. However, modifiers of *GBA* penetrance have not yet been fully elucidated.

**Methods:**

We characterized the transcriptomic profiles of circulating monocytes in a population of patients with PD and healthy controls (CTRL) with and without *GBA* variants (*n* = 23 PD/GBA, 13 CTRL/GBA, 56 PD, 66 CTRL) and whole blood (*n* = 616 PD, 362 CTRL, 127 PD/GBA, 165 CTRL/GBA). Differential expression analysis, pathway enrichment analysis, and outlier detection were performed. Ultrastructural characterization of isolated CD14+ monocytes in the four groups was also performed through electron microscopy.

**Results:**

We observed hundreds of differentially expressed genes and dysregulated pathways when comparing manifesting and non-manifesting *GBA* mutation carriers. Specifically, when compared to idiopathic PD, PD/GBA showed dysregulation in genes involved in alpha-synuclein degradation, aging and amyloid processing. Gene-based outlier analysis confirmed the involvement of lysosomal, membrane trafficking, and mitochondrial processing in manifesting compared to non-manifesting *GBA*-carriers, as also observed at the ultrastructural levels. Transcriptomic results were only partially replicated in an independent cohort of whole blood samples, suggesting cell-type specific changes.

**Conclusions:**

Overall, our transcriptomic analysis of primary monocytes identified gene targets and biological processes that can help in understanding the pathogenic mechanisms associated with *GBA* mutations in the context of PD.

**Supplementary Information:**

The online version contains supplementary material available at 10.1186/s13024-022-00554-8.

## Background

Mutations of the *GBA* gene, encoding beta-glucocerebrosidase (GCase), have long been recognized as the major genetic risk factor for Parkinson’s disease (PD) [1–4]. Mono- and biallelic mutations of *GBA* can increase the risk of developing PD up to 10 times compared to the general population, with an incidence ranging from 2 to 30% across different ancestries [5]. More than 60 pathogenic variants of *GBA* have been identified in PD, where the N370S and L444P mutations account for up to 70–80% of those [6–8]. The molecular mechanisms mainly affected in GBA-PD that have been reported so far encompass the endo-lysosomal pathways, vesicular trafficking, lipid metabolism, and the cell stress response [9–17]. Different hypotheses have also been suggested to explain the relationship between *GBA* variants, reduced GCase activity, and alpha-synuclein accumulation, one of the hallmarks of PD [10, 14, 16]. However, because of the incomplete penetrance of the variants of this gene, it is still not clear whether additional modifiers are responsible for the onset of PD in some of the carriers. The identification of these possible modifiers is crucial for targeted therapeutic interventions or to be leveraged as diagnostic biomarkers. In the literature, a possible modifier effect on *GBA* has been reported for variants in the cathepsin B (*CTSB*) and alpha-synuclein (*SNCA*) genes, common variants in the proximity of the *GBA* gene, GBA pseudogene 1 (*GBAP1*), Metaxin 1 (*MTX1*) and Bridging Integrator 1 (*BIN1*) genes, as well as the leucine repeat rich kinase 2 (*LRRK2*), the other major genetic risk factor of PD [18–24].

*GBA* is ubiquitously expressed across tissues. Mutations of this gene have been associated with aberrant monocyte/macrophage-mediated inflammatory response in the periphery, and with microglia activation in the brain of transgenic animal models [25–27]. Glial activation appears to start early in the presence of *GBA* variants, as it was reported in asymptomatic carriers of the mutation [28]. Since the initial report in 1998 of activated microglial cells in brains of subjects affected with PD, further evidence has stressed a possible role of inflammation in PD pathogenesis involving the peripheral and the central immune response [3, 29–35]. Genetic studies also suggest a link between PD and the immune response [36–40]. Indeed, recent genome-wide association studies (GWAS) identified a number of loci associated with PD which are in close proximity with genes related to the immune and inflammatory response [38]. In adjunct, a polarization of the cis-regulatory effect of common variants associated with PD was identified in the innate immune compartment compared to the adaptive response [39]. By assessing the transcriptomic profiles of CD14+ monocytes and microglia cells from a large cohort of subjects with PD and healthy controls we were able to describe a distinctive expression profile in these cells, identifying dysregulation of genes in the lysosomal and mitochondrial pathways [41].

**Fig. 1:**
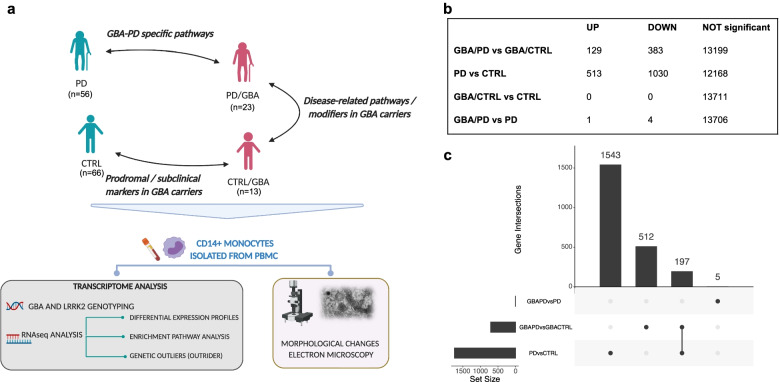
Project design schematic representation and monocytes DEG. **a** Schematic representation of project design and rationale for the comparison of the selected cohorts and analysis of biological samples in monocytes. **b** Number of DEG across groups in monocytes (UP = upregulated genes, DOWN: downregulated genes) (FDR < 0.05). **c** Upset plot summarizing the number of differentially expressed genes at FDR < 0.05 in monocytes between manifesting and non-manifesting *GBA* carriers, PD and CTRL subjects in monocytes and the overlapping genes

Because of the described role of the immune system in the pathogenesis of PD and the important role of *GBA* in these cells, monocytes can represent an informative cell-type to assess the role of this mutation in PD [42, 43]. With the present work we characterized the transcriptomic profiles of isolated CD14 + CD16- monocytes in a cohort of PD patients and controls with and without *GBA* mutations and compared its results with expression data from whole blood generated from a validation cohort (Parkinson’s Progression Markers Initiative - PPMI) (Fig. 1a). We reported a prominent involvement of lysosomal, membrane trafficking, and mitochondrial targets in manifesting vs non-manifesting *GBA*-carriers. Distinctive profiles related to alpha-synuclein-, amyloid- and aging-related processes were detected in manifesting *GBA*-carriers compared to subjects with PD without *GBA* variants. Electron microscopy analysis supported the presence of ultrastructural changes in monocytes from manifesting *GBA*-carriers.

## Material and methods

### Clinical centers and recruitment strategies

Subjects participating in the study were enrolled for the New York Movement Disorder (NYMD) cohort at The Marlene and Paolo Fresco Institute for Parkinson’s and Movement Disorders at the New York University Langone Health (New York), the Bendheim Parkinson Movement Disorders Center at Mount Sinai (BPMD), the Alzheimer’s Research Center (ADRC) and at the Center for Cognitive Health (CCH) at Mount Sinai Hospital (New York). Each Institution’s Institutional Review Board approved the study protocol and the related procedures for subject recruitment, as well as data and samples collection. Subjects were enrolled only upon signing IRB approved informed consent. Enrolled subjects were between the age of 18 and 100 years. The diagnosis of PD was established by qualified movement disorder specialists according to the United Kingdom Parkinson’s Disease Society Brain Bank Clinical Diagnostic Criteria [44]. Healthy controls (CTRL) were defined as aged and gender-matched non-affected subjects, who didn’t have a known diagnosis or evidence of PD or other neurological conditions at the time of enrollment. Non-affected subjects were enrolled mostly among participants’ partners and family members.

The study population of PD and CTRL subjects with and without *GBA* mutations was then selected for the study of the transcriptomic profiles of CD14+ monocytes, based on the availability of blood samples, good quality of extracted RNA and sequencing, according to procedures detailed above, as well as self-reported Northern European ancestry, in order to limit variability due to genetic background architecture related to ancestry. The final population consisted of 56 idiopathic PD (PD - subjects with PD and without *GBA* mutations), 66 CTRL (non-manifesting subjects without *GBA* pathogenic variants), 23 PD/GBA (subjects with PD and *GBA* pathogenic variants), and 13 CTRL/GBA subjects (non-manifesting subjects with *GBA* pathogenic variants) (Supplementary Table 1). Demographic variables are reported in Supplementary Table 1 and they were accounted for in the downstream normalization of the expression data. The great majority of the selected subjects presented overlap with European ancestry, equally distributed between Ashkenazi Jewish (AJ) and non-AJ ancestry (Supplementary Fig. 1a-b).

### Parkinson progressive markers initiative cohort

Data for the Parkinson Progressive Markers Initiative (PPMI) were downloaded from LONI in January 2021. The PPMI is an international, multicenter, observational study collecting clinical and biological data from subjects with PD, non-affected subjects and cohorts at risk for PD aiming to identify clinically significant biomarkers for the care and diagnosis of PD. Enrolled subjects with PD are de novo patients, initially recruited within 2 years from diagnosis. Subjects with PD and controls and known genetic mutations associated with PD, such as *GBA*, *LRRK2*, and *SNCA* are enrolled in the genetic cohort or genetic registry. An extensive description of the PPMI study and collected data and information can be found on www.ppmi-info.org. For this study we selected only subjects with a diagnosis of Parkinson’s disease and non-affected control subjects, with and without mutations of the *GBA* gene. After removing subjects with missing data that would interfere with downstream analysis and considering only baseline visit, the final cohort consisted of 127 PD/GBA, 165 CTRL/GBA, 616 PD and 362 CTRLs.

### Sample collection and processing

Blood samples were collected fresh on the day of the research visit, in the morning, to reduce the variability of sample components and cell activation. Samples were collected in Vacutainer blood collection tubes with acid citrate dextrose (ACD) (BD Biosciences). Samples were processed within 2–3 hours from collection.

DNA was extracted from whole blood (0.5 ml) using the QiAamp DNA Blood Midi kit (Qiagen) according to the manufacturer’s instructions. Nanodrop was utilized to assess DNA quality and concentration.

Sample processing consisted in isolation of peripheral blood mononuclear cells (PBMC) and subsequent CD14+ monocytes purification. For PBMC isolation, SepMate tubes (StemCell Technologies) were used. After dilution in 2-fold PBS (Gibco) tubes were filled with 15 ml of Ficoll-Plaque PLUS (GE Healthcare) and centrifuged at 1200 g for 10 mins, followed by wash with PBS. Monocyte isolation was performed through sorting of 5 million PBMCs utilizing the AutoMacs sorter with CD14+ magnetic beads (Miltenyi) according to manufacturer’s instructions. Sorted monocytes were stored at − 80 °C in RLT buffer (Qiagen) + 1% 2-Mercaptoethanol (Sigma Aldrich). Isolated monocytes stored in RLT buffer were first thawed on ice. RNA was isolated with the RNeasy Mini kit (Qiagen) according to manufacturer’s instructions, including the DNase I optional step. RNA was then stored at − 80 °C until library preparation. RNA integrity number (RIN) was assessed with TapeStation using Agilent RNA ScreenTape System (Agilent Technologies). RNA concentration was obtained with Qubit.

For the PPMI cohort, blood samples were centrally collected and processed as reported by the study’s related documentation (www.ppmi-info.org) and as previously reported [45]. Cell type composition (percentage of neutrophils, lymphocytes, monocytes, basophils, eosinophils) was provided as well with metadata.

### Sample genotyping

Genotyping of the DNA of the samples was performed with the Illumina Infinium Global Screening Array (GSA). This consists of a genome-wide backbone of 642,824 common variants and custom disease SNP content of about 60,000 SNPs. Screening for the most common genetic mutations of the *GBA* and *LRRK2* genes associated with Parkinson’s disease and more frequent among the Ashkenazi Jewish ancestry was performed through targeted genotyping at Dr. William Nichols’ laboratory at the Cincinnati Children’s Hospital. In particular, for the *LRRK2* gene the G2019S variant was screened; for the *GBA* the following 11 variants were analyzed: IVS2 + 1, 84GG, E326K, T369M, N370S, V394L, D409G, L444P, A456P, R496H, RecNcil. The percentage of each mutation across the entire population and within manifesting and non-manifesting carriers was calculated for both *LRRK2* and *GBA* mutations.

### RNA sequencing

Part of the cohort were processed in house for RNA library preparation. TruSeq Stranded Total RNA Sample Preparation kit (Illumina), with the Low Sample (LS) protocol, was utilized for library preparation according to the manufacturer’s instructions. For the rest of the samples RNA-seq libraries was prepared by a commercial service (Genewiz Inc.). RNA was shipped and processed according to a standard RNA-seq protocol. The Ribo-depletion strategy to remove rRNA was utilized for both samples processed in house and at Genewiz Inc. All samples were sequenced at Genewiz Inc. on an Illumina HiSeq 4000 platform with 150-bp paired-end reads and an average of 60 million reads per sample. Sequencing was performed in four independent batches. For isolated CD14+ monocytes, RNA-seq data were obtained from 56 PD, 23 PD/GBA, 66 CTRL, and 13 CTRL/GBA subjects.

For the PPMI cohort, RNA was sequenced at Hudson Alpha’s Genomic Services Lab on an Illumina NovaSeq6000. As reported by the PPMI consortium, rRNA+globin reduction and directional cDNA synthesis using the NEB kit were performed. Samples were prepped using the NEB/Kapa (NEBKAP) based library prep, following second-strand synthesis. Sequencing was performed on the Illumina 6000 platform, generating on average 100 million 125 bp paired reads per sample.

### Genotyping and ancestry analysis

Global Screening Array (GSA) was used to genotype each individual. The following quality control metrics were applied: minor allele frequency (MAF) > 5%, SNP and samples call rate > 95%, Hardy-Weinberg equilibrium (HWE) *P*-Value > 1 × 10–6. PLINK was utilized to identify and remove duplicated/related samples using pairwise IBD (identity-by-descent) estimation (PLINK PI_HAT values 0.99–1).

Genetic ancestry of samples were confirmed through principal component analysis [46] and comparing multidimensional scaling (MDS) of the values of the study cohort with data from the Phase 3 of 1000 Genome Project samples. For the Ashkenazi Jewish (AJ) only, analyses were repeated using a custom panel as a reference (based on [47]).

### Expression data normalization

FASTQ files were processed with the RAPiD pipeline [48] implemented in the NextFlow framework (RAPiD-nf) (“Nextflow - A DSL for Parallel and Scalable Computational Pipelines” n.d.) providing automated alignment, quantification, and quality control of each RNA-seq sample. To assess quality of the sequences and technical metrics, SAMtools (v1.9) and Picard (2.20) (“Picard Tools - By Broad Institute” n.d.) were utilized prior to and after alignment with FASTQC (0.11.8) (“Babraham Bioinformatics - FastQC A Quality Control Tool for High Throughput Sequence Data” n.d.) [49]. Then, reads were processed with trimmomatic (v0.36) for adapter trimming [50]. Afterwards, upon creating indexes from GENCODE (v30) (“GENCODE - Human Release 30” n.d.), STAR (2.7.2a) was utilized for aligning the samples to the human reference genome hg38 build (GRCh38.primary_assembly) [51]. Quantification of gene expression was obtained with RSEM (1.3.1) [52]. Quality control metrics were visualized with MultiQC and gene expression results were generated as raw counts, Counts Per Million (CPM), Transcripts Per Million (TPM), and TMM-voom. The following thresholds were used for initial filtering of the data at the sample level: > 20% of reads mapping to coding regions, > 20 Million aligned reads, and ribosomal rate < 30%. Sex mismatch was assessed by comparing reported sex with the expression of genes *UTY* and *XIST*, which didn’t identify any sex mismatch in our cohort. At the gene level, genes with < 1 count per million in at least 30% of the samples were considered low expression genes and were excluded from the downstream analysis. The above processing led to a total of 13,711 genes that were used in all downstream analyses. Variance partitioning of each including surrogate variable (SVs) (explained in paragraph “Linear models for data regression”) showed an average residual less than 25% **(**Supplementary Fig. 2a). Batch effects strongly associated with gene expression variation **(**Supplementary Fig. 2c), that was mitigated after SVs regression (Supplementary Fig. 2b, d).

For the PPMI cohort, transcriptome alignment and quantification were performed by the Accelerating Medicines Partnership (AMP)-PD consortium. FASTQ files were aligned using STAR v2.6.1d to the GRCh38 human genome build. Gene quantification was performed using Salmon v0.11.3 using GENCODE v29 annotations. Count per million (CPM) were calculated using edgeR package, and genes with CPM lower than 1 in more than 70% (of a total of 8322 samples from PPMI, PDBP, and BioFIND studies) were removed (total of 18,123 remaining genes). Next, only baseline PPMI samples (*n* = 1270) were selected for downstream analysis (only considering patients with PD and control subjects).

### Data normalization and covariate selection

For differential expression analysis, gene counts were scale-normalized by the TMM method using edgeR R package and voom transformed using limma R package [53]. Different designs accounting for the majority of available technical and phenotypic variables (rna_batch + Sex + PCT_USABLE_BASES + PCT_RIBOSOMAL_BASES + AJ_gsa_assignment + RIN + PCT_CODING_BASES + PCT_INTERGENIC_BASES + MEDIAN_5PRIME_BIAS + TOTAL_READS + PF_ALIGNED_BASES + PF_MISMATCH_RATE + C1 + C2 + C3 + C4 + C5 + C6 + C7 + C8 + C9 + C10 + PCT_INTRONIC_BASES + PCT_ADAPTER + next_day + C1_AJ + C2_AJ + C3_AJ + C4_AJ + C5_AJ + C6_AJ + C7_AJ + C8_AJ + C9_AJ + C10_AJ + MEDIAN_CV_COVERAGE + PCT_ADAPTER + Diagnosis) were tested. However, principal component and MDS analysis showed the persistence of samples outliers with an impact on the downstream analysis. Therefore, to reduce error rate and increase reproducibility of the data, these were then processed with the ‘sva’ R package for Surrogate Variable Analysis [54]. This package allows the identification of surrogate variables to be built directly from a high-dimensional dataset. We estimated 13 surrogate variables while preserving effects from genetic status (presence of *GBA* mutations) and phenotype (subjects with a diagnosis of Parkinson’s disease or control groups). Surrogate variables were built in the design for linear regression. The contribution of known technical and phenotypical variables to the surrogate variables was obtained by linear regression between the surrogate variables and the covariates file and visualized in a heatmap (Supplementary Fig. 2c).

For the PPMI cohort, the following variables were regressed for downstream analysis: Interaction + age_at_baseline + sex + rin_value + pct_mrna_bases_picard + pct_intergenic_bases_picard + PC1 + PC2 + PC3 + PC4 + PC5 + median_insert_size_picard + plate. Interaction term accounted for genetic status (*GBA*) and disease status (PD vs CTRL).

### Differential expression analysis based on interaction between genetic and disease status

A list of differentially expressed genes was obtained with the limma package in R by combining expression data (after TMM normalization and voom transformation in R) and surrogate variables. R package limma version 3.38.3 was used to fit a linear model and provide *P*-value upon performing Bayesian moderated t-test. Multiple testing correction with Benjamini-Hochberg False Discovery Rate (FDR) was obtained leveraging the function in the limma package. The cohort of subjects was subdivided into subgroups based on the disease status (subjects with PD vs controls (CTRL)) and *GBA* genetic mutation status (subjects carrying at least one *GBA* variant (PD/GBA, CTRL/GBA) and subjects with no *GBA* variants (PD, CTRL).

We utilized a nested model to analyze expression data in reference to the variable of interest, which consisted in disease status and GBA-mutation status. In addition, to explore the effects of the GBA mutation on the disease status, an interaction term was added to the design as follows: [(PD/GBA – CTRL/GBA) – (PD – CTRL)]. Results from the comparison of each pair of groups were then extracted. A threshold of FDR < 0.05 was utilized as a threshold of significance.

### Gene set enrichment analysis

Gene set enrichment analysis was performed utilizing the set of differentially expressed genes from the nested interaction model analysis considering genes with FDR < 0.05. Enrichment analysis was performed separately for upregulated and downregulated genes in order to better characterize our set of differentially expressed genes. Gene Set Enrichment Analysis (GSEA) was used for the analysis of different terms from the Gene Ontology (GO) list (specifically: Cellular Component (CC), Molecular Function (MF), and Biological Processes (BP)) [55]. Gene-set enrichment with FDR < 0.01 or 0.05 (as specified in the results) were considered. Filters were set for gene-sets with less than 2000 genes. We analyzed up to the first 20 significant enriched terms.

For the dataset obtained from analysis with OUTRIDER [56] tool (see below) the additional following tools were utilized:

1. g-profiler (https://biit.cs.ut.ee/gprofiler/gost), a web server for functional enrichment analysis. Input data were the list of up and downregulated genes separately.
2. Ingenuity pathway analysis (IPA). Canonical data analysis for gene-set enrichment was performed. Statistically significant enriched terms with *P*-value < 0.05 were accounted for in the final results.

The results from the different tools were then combined together based on *P*-values after multiple corrections.

### Curated gene-set analysis

Gene-set enrichment analysis was also performed to assess the enrichment in our sets of differentially expressed genes of curated pathways and gene-sets relevant to previously reported pathways related with GBA functions and PD mechanisms. These encompassed genes involved in the following pathways (Supplementary Table 2): lysosomal database: 435 genes from The Human Lysosome Gene Dataset; lysosomal storage disease causative gene (LSD list) (54 genes) classified as sphingolipidoses, neuronal ceroid lipofuscinosis, mucolipidosis/oligosaccharides diseases; mitochondrial gene list from [57] (315 genes), classified in distinct mitochondrial pathways as reported in the cited paper, such as mitonuclear cross-talk, mitochondrial dynamics, and OXPHOS; ubiquitin-related gene list (428 genes) from ubiquitin-like modifier activating enzymes and ubiquitin conjugating enzymes E2 (HUGO Gene Nomenclature Committee (HGNC) dataset), and ubiquitin ligase E3. We also assessed targeted enrichment for pathways identified by specific GO and involved in vesicle trafficking and the endolysosomal pathways (based on terms “membrane”, “lysosome”, “endocytosis”, “exocytosis”, that identified 32 pathways, (based on terms “membrane”, “lysosome”, “endocytosis”, “exocytosis”, that identified 32 pathways, Supplementary Table 3). and the following additional pathways: NOTCH1 signaling pathway GO:0007219; senescence associated vacuoles: GO:0010282 (plant); cell signaling via exosome: GO:0099156; cellular senescence: GO:0090398; lipid storage: GO:0019915, GO:0006869; lipid transport GO:0032594; tau protein binding GO:0048156; regulation Tau kinase activity GO:1902947, GO:1902949, GO:1902948; Golgi related pathways: GO:0048211, GO:0005795, GO:0005794, GO:0005796, GO:0051645, GO:0006895, GO:0035621, GO:0055107, GO:0006888. Fisher exact test was run to assess the enrichment of curated terms in the differential expressed gene lists.

### Genetic outliers

RNA-seq data can be also used to identify expression outliers within each single sample that may be expression of underlying genetic mutations, especially in regulatory regions, or compensatory/deregulated mechanisms. Different tools have been reported in the literature to explore this approach, based on Z-score distribution or a combination of Z-scores and the negative binomial distribution, respectively [58, 59]. These tools presented some limitations such as the lack of specific statistical tests to compare the expression data and the lack of regression for known and unknown covariates that can greatly affect gene expression profiles. OUTRIDER is an additional tool that, instead, utilizes autoencoders to control for variation linked to unknown factors for data normalization. Single genes and single individuals outliers are then detected by comparing univariate cases with the distribution of each gene across the population, by calculating the negative binomial distribution of each single sample compared to all samples [56]. Autoencoders are also discharging samples with an excess of outlier genes that may be related to other causes than having a biological relevance [56].

Count per million > 1 in more than 30% of the samples were implemented in the tools. Data were normalized leveraging autoencoders (“OUTRIDER - OUTlier in RNA-Seq fInDER”). Normalized dispersion and mean were then fitted in a binomial model followed by computation of two-sided *p*-value. The significance threshold was set at an FDR adjusted *P*-value cut-off of 0.05 and z-score threshold of 2.

### Ultrastructural characterization of CD14+ monocytes

CD14+ monocytes were isolated fresh, within 3 hours from collection, from PBMCs as described above. Isolated monocytes were pelleted (*n* = 2 per group) from manifesting (PD-GBA), non-manifesting (CTRL-GBA) *GBA* carriers, non-carrier controls (CTRL) and idiopathic PD (PD). Pellets were fixed (2% glutaraldehyde and 2% paraformaldehyde/ 0.1 M Sodium Cacodylate buffer) for 1 week and Epon resin embedded. Ultrathin sections (75 nm) were collected onto 200 mesh copper grids using a Leica Em UCT ultramicrotome, counter-stained with uranyl acetate/lead citrate, and imaged using a Hitachi 7500 TEM equipped with an AMT digital camera. Images were sized and adjusted for brightness and contrast. Transmission electron microscopy images were acquired for 2–3 cells per field for a total of 15 cells for each sample. Sections were evaluated blinded. Qualitative assessment of the following parameters was assessed and compared among the four different groups: mitochondrial shape, endoplasmic reticulum, lysosomes, nucleus, chromatin, vacuoles, vesicles and inclusions.

### Statistical analysis and graphic representation

For comparison of the different parameters of the ultrastructural characterization between the four groups all analyses were performed with GraphPad Prism version 9.1.0 (GraphPad Inc. La Jolla CA, USA). One-way ANOVA between mean of counts from each sample was performed. Data are presented as Mean and SEM.

For transcriptomic analysis, sample size and statistical methods are reported in the legends of each figure. In the figures, asterisks indicate significant adjusted *p*value (* = adjusted *p*value < 0.05, ** = adjusted *p*value < 0.01, *** = adjusted *p*value < 0.001).

Bioinformatic analysis were performed with R (version ​​3.6.0) and graphic representation with R Studio version 1.2.1335. Figure 1a was created with BioRender.com.

## Results

### Phenotype and genetics drive different transcriptomic profiles in monocytes

We collected a cohort of subject with PD/GBA (*n* = 23), PD (*n* = 56), CTRL/GBA (*n* = 13), and CTRL (*n* = 66) (Fig. 1a), of European ancestry (Supplementary Fig. 1a, Supplementary Table 1). Differentially expression analysis of isolated CD14 + CD16- monocytes showed a high number of DEG in subjects with GBA/PD compared to GBA/CTRL (*n* = 512) and in PD compared to CTRL (*n* = 1543) (FDR < 0.05) (Fig. 1b,c). We found a more limited number of DEG between subjects with PD (with and without *GBA* variants, n = 5) and no genes between CTRL/GBA and CTRL (Fig. 1b, c). The comparison of the transcriptomic profiles in monocytes from PD vs CTRL was previously explored in our recent work [41]. Here we will focus on the analysis of the subgroup of patients stratified by *GBA* variants.

Interestingly, expression levels of the *GBA* gene didn’t show any significant differences across the four groups in monocytes (Supplementary Fig. 3a-b). However, lower expression levels of *GBA* were observed in carriers of *GBA* variants considered severe, such as the 84GG and V394L, although considering the limited number of subjects per each group (84GG: PD = 2, CTRL = 2; V394L: PD = 1, CTRL =1; L444P/A456P/RecNciI: PD = 1, CTRL = 0) (Supplementary Fig. 3a, Supplementary Table 4).

### Exocytosis- and myeloid cell activation related genes are impaired in monocytes of manifesting vs non-manifesting *GBA* carriers

We first characterized the expression profiles of isolated monocytes from manifesting and non-manifesting *GBA* mutation carriers to elucidate disease mechanisms of *GBA*-related PD. Among the 512 DEG, 197 genes overlapped with genes differentially expressed between PD and CTRL subjects with no *GBA* mutations in monocytes (FDR < 0.05) with significance concordance of directionality (R = 0.92, *p* < 0.001) (Fig. 1c, Supplementary Fig. 10a).

**Fig. 2:**
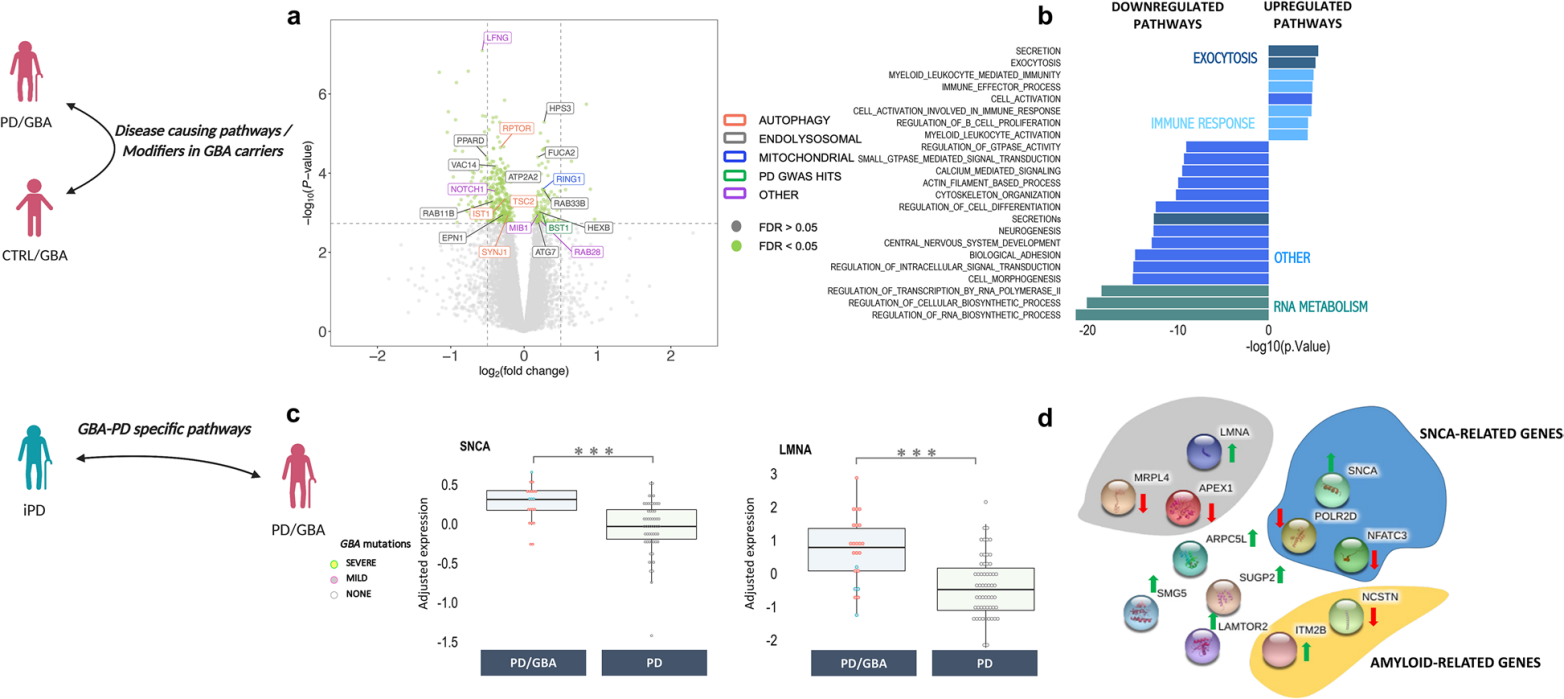
Differential expression profiles between PD/GBA, CTRL/GBA, and PD. **a**-**b** PD/GBA vs CTRL/GBA. **a** Volcano plot represents log_2_ fold change (log2FC) (x-axis) and -log_10_ of *P*-values (y-axis) of the differentially expressed genes between PD/GBA and CTRL/GBA groups. Green dots represent genes with FDR < 0.05. Selected genes were highlighted based on overlap targeted pathways as indicated in the legend on the right side. **b** Pathway enrichment analysis of differentially expressed genes between PD/GBA vs CTRL/GBA subjects. Pathway enrichment of genes differentially expressed between PD/GBA and CTRL/GBA subjects with FDR < 0.05 for GO terms are reported. Light blue: pathways related to the immune response; Dark green: pathways related to exocytosis; Light green: pathways related to RNA metabolism; Dark blue: other pathways. **c**-**d** PD/GBA vs PD. **c** Differential normalized expression count of *SNCA* and *LMNA* between PD/GBA and PD. Disease and genetic status are reported on the x-axes. Each dot represents a subject. Dots are colored based on *GBA* mutations (as reported in the legend: *GBA* mild mutations (N370S, E326K, R496H), *GBA* severe mutations (L444P/A456P/RecNciI, V394L, 84GG, 84GG/T369M, N370S/RecNciI)). Asterisks indicate significant adjusted *p*-value (* = adjusted *p*-value < 0.05, ** = adjusted *p*-value < 0.01, *** = adjusted *p*-value < 0.001; statistics: Mann-Whitney U test). **d** Schematic representation (STRING, [150]) of functionally relevant genes differentially expressed between PD/GBA and PD cohorts. Genes are grouped in colored circles based on shared functional pathways (yellow: amyloid-related genes, blue: SNCA-related genes, grey: PD hits related genes). Arrows indicate whether genes are up (green) or down (red) regulated in the PD/GBA vs the PD cohort

Gene-set enrichment analysis (GSEA) of differentially upregulated genes showed enrichment of pathways related to the myeloid compartment activation, exocytosis, and secretion (FDR < 0.05) (Fig. 2b). Downregulated genes were enriched for transcription/RNA-metabolism related pathways, signal transduction (synapses and calcium mediated signal transmission), kinase activity, and vesicle secretion (Fig. 2b).

Taking a set of curated gene lists and gene ontology (GO) pathways related to membrane trafficking, exocytosis, lysosomal, ubiquitin, and proteasomal pathways, we observed a significant enrichment only for the vesicle membrane pathway within the set of significantly downregulated genes (Fisher’s exact test; adjusted *P*-value < 0.05), which included the *ATP2A2*, *RIPOR1*, *SYNJ1* genes (Supplementary Table 2–3). No significant enrichment was identified for the membrane trafficking-related genes from previously prioritized genes in PD, as reported in Bandres-Ciga et al., 2019 [9].

Interestingly, significant DEG included targets implicated in monogenic forms of PD (such as *ATP13A2* and *LRRK2*), Tau-related pathways, senescence (*FUCA2* and *HEXB)*, *NOTCH1*-related genes (a highly conserved transmembrane domain protein that is involved in different cellular processes such as cell proliferation, differentiation, apoptotic processes, and modulation of the secretome dynamics in cells), autophagy (such as *RPTOR*), the endolysosomal and mitochondrial pathways, as well as genes associated with PD-GWAS loci (Fig. 2a, Supplementary Fig. 4a-b).

### Aging- and alpha-synuclein-related pathways are impaired in PD/GBA vs PD

When comparing expression profiles in isolated monocytes of subjects with PD with vs without *GBA*-variants, the number of significantly DEG was more limited, including 5 genes at FDR < 0.05 (1 upregulated and 4 downregulated) (Supplementary Fig. 5a). Pathway enrichment analysis was not possible due to the small number of DEGs between these two groups. Considering the small sample size of these two groups, we also looked at genes with sub-threshold expression levels (FDR < 0.15; 44 genes). Interestingly, in PD/GBA subjects there was an upregulation of the alpha-synuclein gene (*SNCA*) and related genes (i.e. *POLR2D* and *NFATC3)* compared to PD (FDR < 0.15) (Fig. 2c-d, Supplementary Fig. 5b) [60]. We also identified genes related to the amyloid pathways (*ITM2B* and *NCSTN* genes), and aging process, such as *LMNA,* which is responsible for the progeria syndrome [61] (Fig. 2b). Finally, we found a deregulation of *LAMTOR2*, an amino acid sensing and activator of mTORC1 through its recruitment in the lysosomes (Fig. 2b).

Taken together, these observations suggest a deregulation of pathways associated with alpha-synuclein, aging and PD-related genetics in the PD/GBA vs PD group.

Finally, a small set of genes was significantly differentially expressed in the PD/GBA group compared to both PD and CTRL/GBA subjects (*n* = 6, FDR < 0.15), and included *FILIP1L* [62], and *HPS3* (Supplementary Fig. 6). *HPS3* is particularly interesting in this context since this gene encodes for a subunit of the lysosome-related organelles complex-2. Mutations of this gene are responsible for the Hermansky-Pudlak Syndrome 3, a systemic condition affecting the immune system and melanin, similarly to Chediak-Higashi syndrome (CHS). CHS has been previously associated with PD [63].

### Gene expression outliers highlight targeted pathways in manifesting and non-manifesting carriers

We then performed gene expression outlier analysis. This tests for genes with significantly different levels of expression in a single individual compared to the rest of our cohort. Gene expression outlier individuals may harbor rare genetic variants in those genes [56]. In our cohort, we detected 493 outlier genes and 125 subjects (out of 158) with at least one outlier gene after normalization (Supplementary Fig. 7a-c).

**Fig. 3:**
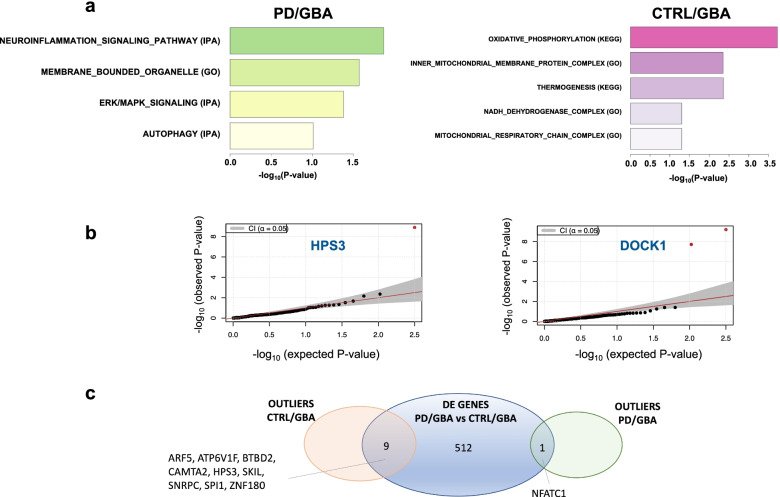
Enrichment analysis of outlier genes in the four cohorts (PD/GBA, CTRL/GBA, PD, CTRL). **a** Pathway enrichment analysis of outlier genes in the PD/GBA and CTRL/GBA cohort (based on GSEA, IPA and g-profiler tools). Bars represent *p*-value (−log_10_(*p*-value)). **b** Scatter plot representing -log_10_ (p-value) of two of the outliers genes identified in previous analysis. **c** Venn-diagram representing overlapping genes between differential expression analysis and outliers analysis in manifesting and non-manifesting carriers in isolated monocytes

Consistent with previous analysis, pathway enrichment analysis of the genetic outliers of subjects in the PD/GBA group confirmed a significant enrichment of pathways related to neuroinflammation (including *ICAM1*, *NFATC1*, *IRAK4*), membrane bounded organelle (such as *VPS41*), ERK/MAPK signaling, and autophagy (such as *DOCK1* - involved in cytoskeletal rearrangement for phagocytosis of apoptotic cells and mobility of the cells) (Fig. 3a-b).

Instead, outlier genes in the CTRL/GBA group were mostly related to the mitochondrial functions: oxidative phosphorylation, inner mitochondrial membrane protein complex, thermogenesis, NADH dehydrogenase complex, and mitochondrial respiratory chain complex I. Outliers genes in the enriched pathways included: *MT-ATP* (mitochondrial complex 5), *MT-ND, MT-ND2, MT-ND4* (components of the NADH dehydrogenase or mitochondrial complex I) which are all genes encoded by the mitochondrial genome (Fig. 3a, Supplementary Fig. 7). We found 9 outlier genes in CTRL/GBA subjects and one outlier gene in PD/GBA (*NFATC1*) subjects that overlapped with the DEG between PD/GBA and CTRL/GBA (Fig. 3c, Supplementary Fig. 7).

### Whole blood transcriptomic profiles only partially reflect the findings in isolated CD14+ monocytes

**Fig. 4:**
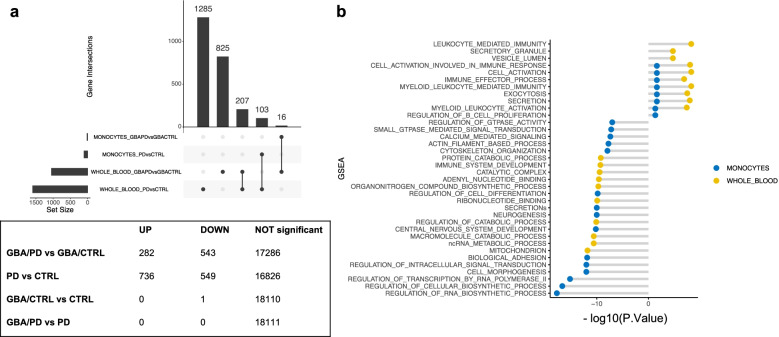
Differential expression profiles in whole blood and overlap with monocytes data. **a** Upset plot summarizing the number of differentially expressed genes at FDR < 0.05 in whole blood between manifesting and non-manifesting *GBA* carriers, and PD and CTRL subjects and the overlapping genes with monocytes. **b** Number of DEG across groups in whole blood (UP = upregulated genes, DOWN: downregulated genes). **c** Dysregulated pathways in CD14+ monocytes and whole blood in GBA/PD vs GBA/CTRL. The plot represent the -log_10_(*P* Value) the enriched pathways in the differentially up-regulated and down-regulated genes when comparing GBA/PD vs GBA/CTRL in both isolated CD14+ monocytes (blue) and whole blood (yellow) at FDR 5%. The figure shows an overlap in monocytes and whole blood of the upregulated pathways related to membrane trafficking and immune response

We then assessed the overlap between the transcriptomic profiles in our monocytes cohort with an independent cohort of whole blood (Fig. 4a, Supplementary Table 5). Monocyte counts and RNA concentration in our cohort and in the PPMI study were not significantly different across the four groups, and therefore should not be accountable for the differences of the transcriptomic profiles (Supplementary Fig. 2 and 9a-b). Instead, whole blood composition showed a significant difference in the number of neutrophils and lymphocytes in *GBA* manifesting vs non-manifesting carriers, which may impact differential analyses of transcriptomic data (Supplementary Fig. 9b).

Although we found a high correlation of mean gene expression levels between monocyte and whole blood (Supplementary Fig. 11), the reproducibility of DEG in the two cohorts was only partial (Fig. 4a).

Interestingly, when comparing PD/GBA vs CTRL/GBA we found overlaps of enriched upregulated pathways related to vesicle trafficking and activation of the myeloid compartment in monocytes and whole blood (Fig. 4b). The number of overlapping DEG between GBA/PD and GBA/CTRL in monocytes and whole blood was limited (*n* = 16), although with a high degree of concordance in direction (Fig. 4a, Supplementary Fig. 10c, Supplementary Table 6, Supplementary Table 7). Target genes that were found to be significantly differentially expressed in monocytes (such as *ATP13A2*, *LRRK2*, and *NOTCH1*) were not differentially expressed in whole blood, suggesting that purified cell population may increase the power for detecting important changes despite smaller cohorts (Supplementary Fig. 12a). Similarly to monocytes, *GBA* was not differentially expressed in whole blood across our four groups (Supplementary Fig. 12b).

When we compared the expression profiles of PD subjects with vs without *GBA*-variants in whole blood, no significantly differentially expressed genes at FDR < 0.15 were detected (Fig. 4a). At a nominal *p*-value < 0.001, some overlapping DEG between monocytes and whole blood were found, including *LMNA* (Supplementary Fig. 12b). Enrichment analysis for differentially expressed genes with nominal *p*-value < 0.01 highlighted pathways related to the immune response and cellular transport, but not to mitochondria and vesicular trafficking (Supplementary Fig. 12c). The number of DEG in subjects with PD with and without *GBA* variants was too limited to perform a pathway analysis.

### Ultrastructural characterization of CD14+ monocytes confirms membrane vesicle impairment in PD and *GBA* carriers

Differentially expressed gene profiles from monocytes and whole blood both suggested a dysregulation of genes involved in the endo-lysosomal pathways and mitochondria. To assess whether this had an impact at a molecular level, the ultrastructure analysis of CD14+ monocytes from our four cohorts were performed (Supplementary Table 7).

**Fig. 5:**
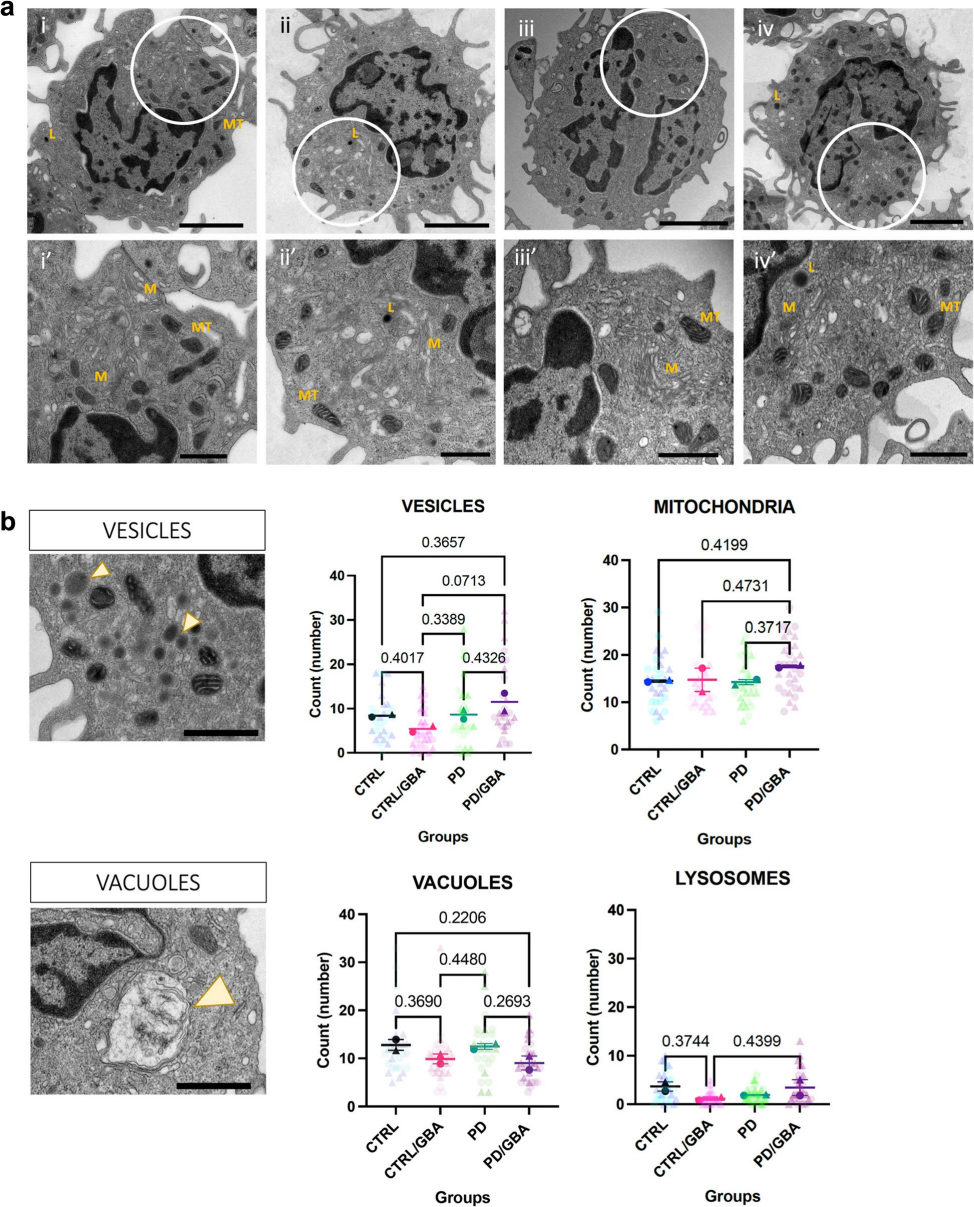
Morphological characterization of CD14+ monocytes. **a** Low magnification (1800X) images (i, ii, iii, iv) and high magnification (4000x) images (i’, ii’, iii’, iv’). Location of the high magnification images within low magnification pictures are highlighted by the white circle in the first row. Scale bar: 2 𝝻m and 800 nm respectively. i/i’) Cells from CTRL and ii/ii’) CTR/GBA subjects showing normal membrane compartment (M, Golgi and Endoplasmic reticulum), lysosomes (L), mitochondria (MT), cell-to-cell adherences, multiple pseudopodal extensions from the cell membrane. iii/iii’) Cells from PD subjects showing highly thickened, and distorted cell membrane compartment (M, Golgi and Endoplasmic reticulum); decreased pseudopodia, RER and free ribosomes; mitochondrial (MT) membranes are severely affected, often lacking external membranous encapsulation and with internally swollen cristae. iv/iv’) Cells from PD/GBA subjects showing small and large vacuoles, normal cell membranes, pseudopodal extensions appear normal, and nuclei. Abnormal membrane assembly (M), ultrastructure, and free ribosomes. **b** Quantification of mitochondria, lysosome, vesicles and vacuoles in four groups (*n* = 2 subjects per group, 15 cells per sample). Example of vacuole and vesicles reported in the figures on the left highlighted by yellow arrowhead (scale bar 800 nm). Data represents count from each cell from the two samples per group, and mean and SEM of the two replicates. *p*-value from one-way ANOVA analysis is reported above each comparison (*p*-value < 0.5 were reported)

Qualitative assessments showed that in the CTRL/GBA and CTRL groups samples showed normal cell size (12–20 μm), with intact cytoplasm organelles (Golgi (G), endoplasmic reticulum (ER)/rough ER, ribosomes, pinocytotic vesicles, lysosomal granules, phagosomes, mitochondria, and microtubules), indented nuclei, and pseudopodia extend from cell surfaces (Fig. 5a, i/i’; ii/ii’).

Images from the PD group showed that the majority of cells were relatively intact. However, subpopulations presenting distinct characteristics were identified. These cells showed impairment of cell membranes, manifesting with enhancing of the cytoplasmic membrane (in the Golgi and ER) and lacking external membranous encapsulation and swelling of internal swollen cristae of the mitochondria (MT) (Fig. 5a, iii/iii’).

In the PD/GBA group, distinct subpopulations of monocytes showed small and large vacuoles, but normal cell membranes, pseudopodal extensions, and nuclei intact. Abnormal membrane-organelles, such as Golgi/ER, RER were severely affected in these cells as well. However, quantification of the number of mitochondria, lysosome, vesicles, and vacuolar inclusions found no statistical difference between the four groups (Fig. 5b).

## Discussion

The involvement of the immune system in the pathogenesis of PD, both of the innate immune system in the periphery and in the brain and the adaptive immune system, has attracted growing attention over the last few years [29–31, 33–35, 39, 64–66]. GCase, the enzyme encoded by the PD-genetic risk factor *GBA*, is important for the metabolic processes of scavenger cells such as monocytes and macrophages. Compared to previous works that performed unbiased transcriptomic analysis comparing PD and CTRL subjects, we report here the first transcriptomic analysis in whole blood and CD14+ monocytes stratified by *GBA* mutation status [41, 45]. We found that expression profiles of patients with PD and *GBA* variants were significantly different from both idiopathic PD patients and non-manifesting carriers (Fig. 1b-c) with only a partial overlap with genes differentially expressed between PD and CTRL subjects without *GBA* mutations, suggesting that specific pathways are altered in the presence of variants of this gene (Fig. 1c).

Deregulated genes and pathways in manifesting *GBA*-carriers highlighted molecular mechanisms previously associated with PD pathogenesis in other cellular or animal models, such as the endo-lysosomal pathway, the mitochondrial pathway, inflammatory markers as well as genes related to monogenic forms of PD and PD GWAS (such as *BST1* and *SNCA*) (Fig. 2a) [38, 67–69]. This suggests that monocytes can mirror many processes reported in cellular and animal models of PD as well as dopaminergic neurons. Interestingly, ultrastructural changes in monocytes may reflect transcriptomic signatures. In subpopulations of cells from GBA/PD subjects, we found impairment of the membrane compartment, including a poor organization of the endoplasmic reticulum and Golgi (Fig. 5). Whether these changes are consistent with an active role of the innate immune compartment in causing the disease or represent just a reactive response cannot yet be inferred from our data, but it would be worth further exploring. Nevertheless, monocytes may represent a good platform to recapitulate and study PD-associated pathogenic mechanisms. Even more so, despite the small number of subjects in our cohorts – which, to the best of our knowledge, still represents the largest transcriptomic profiles analysis in patients with PD carrying mutations of the *GBA* gene - we were able to identify a large number of differentially expressed genes. This suggests the importance of considering purified cell types, such as isolated CD14+ monocytes, to dramatically reduce the variability due to background noise signals, such as in whole blood.

When we compared the expression profiles of subjects with PD with and without mutations of the gene *GBA,* we found an interesting dysregulation of aging-related targets (Fig. 2c-d). The question remains whether aging processes of PD can be further accelerated in carriers of *GBA* mutations or whether the pathogenic variants of this gene are instead responsible in the first place for the activation of pathways that can cause accelerating aging. Interestingly, we also reported that monocytes of subjects with PD and *GBA* mutations showed an increased expression of the *SNCA* gene, the hallmark protein of PD (Fig. 2c). We know that in brains of subjects with *GBA*-related PD there is a robust deposition of this protein, which could explain the more aggressive phenotype in carriers of these mutations compared to idiopathic PD in terms of an earlier age of onset and increased frequency of cognitive impairment and non-motor symptoms [70]. It can be speculated that increased expression levels of *SNCA* can represent a compensatory mechanism to its aberrant accumulation due to decreased GCase activity, as previously described [71, 72]. However, *SNCA* upregulation could also represent a precipitating factor for disease onset in predisposed subjects, such as carriers of mutations of *GBA*.

Only a small subset of genes were differentially expressed in both monocytes and whole blood from manifesting vs non-manifesting *GBA*-carriers (Supplementary Table 6). We hypothesized that monocytes-specific changes can be missed when analyzing multiple cell types at once in whole blood. Indeed, cell type composition in whole blood showed a significant difference in the number of neutrophils and lymphocytes in manifesting vs non-manifesting GBA carriers, that could affect the overall transcription signal (Supplementary Fig. 9b). On the contrary, the expression of other gene sets may be more consistent across cell types, and thus overlap between monocytes and whole blood. Previous works from the literature showed that additional cell types of the innate immune compartment as well as T-cells, are involved in PD (65,81). To further explore these hypotheses it will be worth analyzing the expression profiles in PD and CTRL subjects in different purified cell types.

In our previous work assessing expression profiles of circulating monocytes and microglia in idiopathic PD patients, we identified an opposite deregulation of the mitochondrial signature in the immune cells in the periphery compared to the central nervous system in PD patients [41]. Impaired mitochondrial genes were also detected as outliers among CTRL/GBA subjects, suggesting an involvement and a possible modulatory effect of these genes in the *GBA*-related pathogenic mechanisms as previously suggested (Fig. 3) [73].

One limitation of our study, which is shared with many of the works comparing profiles of manifesting vs non-manifesting carriers of mutations with a reduced penetrance, is the fact that we cannot rule out whether some of the non-manifesting carriers of *GBA* mutations will eventually manifest PD at the end of their life. However, because of our large sample size (considering both monocytes and whole blood) and the reduced penetrance of *GBA*, even if some of the subjects who are currently considered non-manifest carriers will eventually phenoconvert to PD, this will not significantly impact the results of our analysis. In the future, it will be interesting to look for the possible prodromal changes in these subjects through longitudinal analysis.

Our analyzes aimed to leverage immune cells as a platform to identify in a systematic and rigorous manner dysregulated pathways and target genes that can represent potential modifiers of *GBA*-driven PD. Future validation analysis in experimental models (cellular or animal models) will help clarify the contribution of these modifiers in the disease mechanisms. In addition, integration with genome sequencing data may allow us to identify genetic variants that can modulate *GBA* penetrance.

## Conclusions

In conclusion, our results showed that peripheral innate immune cells can be informative in the assessment of disease mechanisms associated with PD. We identified a set of genes and molecular pathways that are specific for *GBA*-related PD, such as a deregulation of SNCA and pathways related to metabolism of beta-amyloid and aging compared to PD, as well as dysregulation of the lysosomal, membrane trafficking, and mitochondrial processing in manifesting compared to non-manifesting *GBA*-carriers (including genes such as *LRRK2*, *ATP13A2*, *BST1* and *NOTCH1*), also confirmed at the ultrastructural level in monocytes. Further investigation will clarify the possible role as disease biomarkers and of these hits.

## Supplementary Information

**Additional file 1: Supplementary Table 1.** Clinical characterization of study cohort. Summary of demographic, clinical and genetic features of the cohort of subjects (PD and CTRL) whose purified CD14+ monocytes were used for integrated genomic analysis.

**Additional file 2: Supplementary Table 2.** Targeted pathway enrichment in PD/GBA vs CTRL/GBA. List of endolysosomal pathways (from GO terms and curated pathways, i.e. ubiquitin pathway) reported in Fig. 5. *p*-value of enrichment as per Fisher exact test of each pathway within the set of up-regulated (UP) and down-regulated (DOWN) genes. Significant enriched pathways (at *p*-value < 0.15) are highlighted in red.

**Additional file 3: Supplementary Table 3.** GO terms pathways enrichment analysis. The table reports the GO terms and ID for the pathways that were tested for enrichment analysis of differentially expressed genes between the CTRL/GBA and PD/GBA cohorts.

**Additional file 4: Supplementary Table 4.** Number of subjects (PD and CTRL) with different *GBA* variants, reported in the text.

**Additional file 5: Supplementary Table 5.** Summary of demographic, clinical and genetic features of the PPMI cohort (whole blood) of subjects (PD and CTRL) in this study.

**Additional file 6: Supplementary Table 6.** Overlapping genes between monocytes and PPMI cohort (whole blood) in manifesting vs non-manifesting GBA-carriers. List of overlapping genes differentially expressed in monocytes and whole blood in manifesting vs non-manifesting carriers. Gene name, description of gene function and logFC and FDR in monocytes and whole blood are reported. In “grey” genes that represent PD-GWAS hits are highlighted.

**Additional file 7: Supplementary Table 7.** Up-regulated and down-regulated pathway related to vesicle and membrane trafficking in isolated CD14+ monocytes and whole blood. The table shows the list of pathways that were found to be deregulated in both isolated CD14+ monocytes and whole blood in the PD/GBA vs CTRL/GBA groups (top), the deregulated genes in each pathway (first column), and the directionality of dysregulation (second and third column). Red “x” indicates genes dysregulated in whole blood, black “x” represents genes dysregulated in isolated CD14+ monocytes.

**Additional file 8: Supplementary Fig. 1.** Characterization of genetic background of donor population. a) Representation of PCA analysis of ancestry of MDS values from the cohort of 158 subjects (PD/GBA, PD, CTRL/GBA, CTRL) compared to 1000 Genome Project samples (Phase 3). The different ancestry are represented in distinct colors (Orange: African; Gold: Ad Mixed American; Green: East Asian; Blue: European; Purple: South Asian; Black: study cohort). B) PCA considering only overlap of MDS values of donor cohort (black) with European ancestry (blue) and AJ ancestry (light blue). **Supplementary Figure 2.** Normalization and quality control of RNA-seq data from isolated CD14+ monocytes. a) Violin plot representing the contribution of each of the surrogate variables (as explained in the text) to the variability of expression data of the study cohort and residual (158 subjects). b) Violin plot representing the contribution of technical, demographical and clinical variables to the variability of expression data of the study cohort and residual (158 subjects). c) Heatmap representing the results of linear regression between the surrogate variables utilized for data normalization and technical variables (from RNA-seq analysis) and metadata. Coefficient of linear regression is reported in the heatmap for each correlation pair. d) Distribution of MDS values of study cohort identified a clear clustering based on batches used for RNA-seq analysis (batches 1 to 4). e) After regression of SVs, variability of MDS values is significantly reduced, with no significant outliers and no clustering based on experimental batches. **Supplementary Fig. 3.** Differential expression of *GBA* in CD14+ isolated monocytes. Box plot representing differential expression levels (normalized expression count) of *GBA* in isolated CD14+ monocytes (a and b). In b) data from isolated CD14+ monocytes of *GBA*-carriers and non-carriers within PD and CTRL subjects were combined and compared. Each dot represents a subject. Dots are colored based on *GBA* mutations (as reported in the legend: *GBA* mild mutations (N370S, E326K, R496H), *GBA* severe mutations (L444P/A456P/RecNciI, V394L, 84GG, 84GG/T369M, N370S/RecNciI)). *p*-value of different expression levels is reported on top (statistics: Mann-Whitney U test). *GBA* variants different from N370S are labeled in the boxplot. **Supplementary Fig. 4.** Differential expression of target genes in manifesting and non-manifesting carriers in monocytes. a) Volcano plot showing differentially expressed genes with FDR < 0.05 (green dots) in isolated monocytes from manifesting vs non-manifesting carriers. Genes related to targeted pathways (*LRRK2*, *ATP13A2*, *NOTCH1* and *TAU*) are highlighted. b) Box plots of differential levels of expression of the targeted genes (*ATP13A2*, *LRRK2*, *NOTCH1*), between manifesting and non-manifesting carriers in isolated monocytes. Each dot represents a subject. Dots are colored based on *GBA* mutations (as reported in the legend: *GBA* mild mutations (N370S, E326K, R496H), *GBA* severe mutations (L444P/A456P/RecNciI, V394L, 84GG, 84GG/T369M, N370S/RecNciI)). Asterisks indicate significant *p*-value (* = *p*-value < 0.05, ** = *p*-value < 0.01, *** = *p*-value < 0.001, statistics: Mann-Whitney U test). **Supplementary Fig. 5.** Differential expression analysis in monocytes in PD patients with and without *GBA*-variants. a) Volcano-plot representing logFC (x-axes) and *p*-value (y-axes, -log_10_ *p*-value) of differential expressed genes between PD/GBA and PD as per nested interaction model. Highlighted in yellow are genes with FDR < 0.15 (44 total genes). ID labels of functionally relevant genes and of genes differentially expressed in whole blood (PPMI overlap of genes at nominal *p*-value <0.001) are reported in the plot. b) Differential normalized expression count of *SNCA* and *LMNA* between PD/GBA and PD, compared to CTRL/GBA and CTRL subjects in isolated CD 14+ monocytes. Asterisks indicate significant *p*-value (* = *p*-value < 0.05, ** = *p*-value < 0.01, *** = *p*-value < 0.001, statistics: Mann-Whitney U test). Disease and genetic status are reported on the x-axes. Each dot represents a subject. Dots are colored based on *GBA* mutations (as reported in the legend: *GBA* mild mutations (N370S, E326K, R496H), *GBA* severe mutations (L444P/A456P/RecNciI, V394L, 84GG, 84GG/T369M, N370S/RecNciI)). *GBA* variants different from N370S are labeled in the boxplot. **Supplementary Fig. 6.** Differential expression profiles across the four cohorts based on diagnosis and genetic status interaction. a) Volcano-plot representing log_2_ fold change (x-axes) and *P*-Value (y-axes, -log_10_ *P*-Value) of differential expressed genes based on diagnosis and genetics interaction between the four cohorts (PD/GBA, CTRL/GBA, PD, CTRL). Genes with FDR < 0.05 are highlighted in blue and labeled with their IDs. Differentially expressed genes encompassed: *ANGPT1* (angiopoietin gene involved in angiogenesis), *FILIP1L* (Filamin A Interacting Protein 1 Like), *AC138028.2* (novel transcript), *SRGAP1* (Slit-Robo GTPase-activating protein 1), *PIK3R5* (Phosphoinositide 3-kinase regulatory subunit 5, responsible for Ataxia with Oculomotor-Apraxia type 3), *HPS3* (Hermansky-Pudlak Syndrome 3 Protein, biogenesis of lysosomal organelle complex 2 subunit 1). b) Box plots representing expression levels (normalized expression count) of one differentially expressed targeted gene according to interaction term (diagnosis and genetics interaction). Disease and genetic status are labeled on the x-axes. Boxes are colored based on disease and genetic status. Each dot represents a subject. Dots are colored based on *GBA* mutations (as reported in the legend: *GBA* mild mutations (N370S, E326K, R496H), *GBA* severe mutations (L444P/A456P/RecNciI, V394L, 84GG, 84GG/T369M, N370S/RecNciI)). Asterisks indicate significant *p*-value (* = *p*-value < 0.05, ** = *p*-value < 0.01, *** = *p*-value < 0.001; statistics: Mann-Whitney U test). **Supplementary Fig. 7.** QC for analysis of outlier genes. a) Normalization based on surrogate variables, as provided by the OUTRIDER script, of a total of 13711 genes (considering only genes with > 30% of total expression). Discrete relevant variables (Diagnosis, batches of RNAseq analysis (rna_batch), GBA mutations and GBA-related genetic status (carriers or non-carriers of GBA mutations), gender (Sex: male (M) and female (F)) are labeled at the top of the heatmap per each subject. b) Bar-plot reporting number of outlier genes per each subject (out of 158 subjects). Highlighted in orange samples with outlier gene count above 0.1%. c) Summary tables: on the left: number of outliers genes per cohort (PD/GBA, CTRL/GBA, PD, CTRL) (493 pairs); on the right: number of subjects per each cohort with at least one outlier gene (125 unique subjects total with at least one outlier gene). **Supplementary Fig. 8.** Scatter plot of selected genes identified through the OUTRIDER method. Scatter plot representing -log_10_ (*p*-value) of outliers genes identified in previous analysis. **Supplementary Fig. 9.** Cell proportion across patient and control cohorts. a) Monocyte counts (x10^6^) in the GBA/PD, PD, GBA/CTRL, and CTRL groups in the cohort of isolated monocytes (NYMD cohort). Each dot represents the cell count of one subject. Cell count was available for 57 subjects in our cohort. Statistical analysis according to the Wilcoxon test is reported for the comparison between the four groups. b) Cell proportion (neutrophils, monocytes, lymphocytes, eosinophils, basophils) within whole blood samples in the four groups (GBA/PD, PD, GBA/CTRL, and CTRL) in the PPMI cohort. Comparison of each cell type percent across the four groups is reported on the box plots on the right side of the figure (statistical analysis: Wilcoxon test). **Supplementary Fig. 10.** Correlation between genes differentially expressed in isolated CD14+ monocytes and whole blood. We compared the directionality of differentially expression genes between a) GBA/PD vs GBA/CTRL and PD vs CTRL in CD14+ monocytes (*n* = 197); b) GBA/PD vs GBA/CTRL and PD vs CTRL in whole blood (*n* = 207); c) GBA/PD vs GBA/CTRL in CD14+ monocytes and whole blood (*n* = 16); d) PD vs CTRL in CD14+ monocytes and whole blood (*n* = 103). **Supplementary Fig. 11.** Correlation between gene expression levels in isolated CD14+ monocytes and whole blood. Genes with expression with more than 1 CPM in 30% of the samples were considered from both cohorts (discovery cohort: isolated CD14+ monocytes (total number of genes: 13711), validation cohort: whole blood - PPMI cohort (total number of genes: 18111)). Spearman correlation between levels of normalized mean gene expression across subjects within each cohort per sub-group of subjects was calculated (R = 0.78 *p* < 0.001). Genes were normalized with TMM and voom, as detailed in the main text. **Supplementary Fig. 12.** Validation in whole blood of differentially expressed genes in monocytes. a) Differential levels of expression of the targeted genes (*ATP13A2*, *LRRK2*, *NOTCH1*, between manifesting and non-manifesting carriers in whole blood from manifesting and non-manifesting GBA-mutation carriers. b) Differential normalized expression count of *SNCA*, *LMNA,* and *GBA* between PD/GBA and PD, compared to CTRL/GBA and CTRL subjects in whole blood. In a) and b) each dot represents a subject. Dots are colored based on *GBA* mutations (as reported in the legend: *GBA* mild mutations (N370S, E326K, R496H), *GBA* severe mutations (L444P/A456P/RecNciI, V394L, 84GG, 84GG/T369M, N370S/RecNciI)). *p*-value of different expression levels is reported on top (statistics: Mann-Whitney U test). c) Pathway enrichment analysis of differentially expressed genes in whole blood between PD/GBA vs PD subjects with *p*-value < 0.01 for GO terms are reported. Dark blue: pathways related to cell transport; Green: pathways related to immune response; Light blue: other pathways.

## Data Availability

The datasets supporting the conclusions of this article (Raw RNA-seq data) are available as part of the Myeloid cells in Neurodegenerative Disease (MyND) study via dbGAP (study accession ID: phs002400.v1.p1) at https://www.ncbi.nlm.nih.gov/projects/gap/cgi-bin/study.cgi?study_id=phs002400.v1.p1. RNA-seq data for Parkinson’s Progression Markers Initiative (PPMI) cohort were obtained from the Accelerating Medicines Partnership program for Parkinson’s disease (AMP-PD) Knowledge Platform. For up-to-date information on the study, https://www.amp-pd.org.

## Abbreviations

ACD: Acid citrate dextrose
ADRC: Alzheimer’s Research Center
AJ: Ashkenazy Jewish
ANGPT1: Angiopoietin 1
AP2A1: Adaptor Related Protein Complex 2 subunit alpha-1
APEX1: Apurinic/Apyrimidinic Endodeoxyribonuclease 1
ARHGAP1: Rho GTPase activating protein
ATG7: Autophagy Related 7
ATP13A2: ATPase Cation Transporting 13A2
ATP2A2: ATPase Sarcoplasmic/Endoplasmic Reticulum Ca2+ Transporting 2
BIN1: Bridging Integrator 1
BP: Biological Processes
BPMD: Bendheim Parkinson and Movement Disorders (Center at Mount Sinai School of Medicine (NY, US))
BST1: Bone marrow stromal cell antigen 1, ADP-ribosyl cyclase 2, CD157
CC: Cellular Component
CCH: Center for Cognitive Health
CHML: CHM Like Rab Escort Protein
CPM: Count per million
CTRL: Control
CTSB: Cathepsin B
DOCK1: Dedicator of Cytokinesis 1
ER: Endoplasmic reticulum
ERLIN1: ER lipid raft associated 1
FDR: False Discovery Rate
FILIP1L: Filamin A Interacting Protein 1 Like
FUCA2: Alpha-L-Fucosidase 2
GBA: Glucocerebrosidase
GBAP: Glucocerebrosidase pseudogene
GCase: Beta-glucocerebrosidase
GO: Gene Ontology
GSEA: Gene Set Enrichment Analysis
GWAS: Genome wide association study
HEXB: Hexosaminidase Subunit Beta
HPS3: Hermansky-Pudlak Syndrome 3 Protein
IRAK4: Interleukin 1 Receptor Associated Kinase 4
IRB: Institutional Review Board
KMT2D: lysine methyltransferase 2D
ICAM1: Intracellular Adhesion Molecule 1
IL-: Interleukin-
IPA: Ingenuity pathway analysis
iPD: Idiopathic Parkinson’s disease
ITM2B: Integral membrane protein 2B
GSA: Global Screening Array
HGNC: HUGO Gene Nomenclature Committee
LAMTOR2: Late Endosomal/Lysosomal Adaptor, MAPK And MTOR Activator 2
LFNG: O-Fucosylpeptide 3-Beta-N-Acetylglucosaminyltransferase
LMNA: Lamin A/C
LRRK2: Leucine-Rich Repeat Kinase 2
LSD: lysosomal storage disorders
MAF: Minor allele frequency
MAP 4: Microtubule Associated Protein 4
MF: Molecular Function
MIB1: Mindbomb E3 Ubiquitin Protein Ligase 1
MRPL4: Mitochondrial Ribosomal Protein L4
mRNA: Micro RNA
MT-ND (2, 4): Mitochondrially Encoded NADH:Ubiquinone Oxidoreductase Core Subunit (2, 4)
mTORC1: Mechanistic target of rapamycin complex 1
MyND: Myeloid cells in Neurodegenerative Disease
NFATC (1, 3): Nuclear Factor of Activated T-cells, cytoplasmic (1, 3)
NIH: National Institutes of Health
NOTCH1: Notch receptor 1
OUTRIDER: OUTlier in RNA-seq fInDER
PBMC: Peripheral blood mononuclear cells
PCA: Principal component analysis
PD: Parkinson’s disease
PDHX: Propionyl-CoA Carboxylase Subunit Beta
PIK3R5: Phosphoinositide 3-kinase regulatory subunit 5
POLR2D: RNA polymerase II subunit D
QC: Quality control
RIN: RNA integrity number
RNA-seq: RNA-sequencing
RPTOR: Regulatory associated protein of MTOR complex 1
RSRP1: Arginine and Serine Rich Protein 1
SNCA: Alpha-synuclein
SNP: Single nucleotide polymorphism
SRGAP1: SLIT-ROBO Rho GTPase activating protein 1
SVA: Surrogate variable analysis
SYNJ1: Synaptojanin 1
TMM: Trimmed Mean of M-values
TPM: Transcripts Per Million
TSC2: Tuberous Sclerosis Complex 2
UTY: Ubiquitously Transcribed Tetratricopeptide Repeat Containing, Y-Linked
XIST: X-inactive specific transcript
