# Supplementary material for: Transcriptome deregulation of peripheral monocytes and whole blood in GBA-related Parkinson’s disease
Source: Mol Neurodegener. 2022 Aug 17;17:52. doi: 10.1186/s13024-022-00554-8 (PMC9386994; doi:10.1186/s13024-022-00554-8)
Supplement: Supplementary file 1 — Additional file 1: Supplementary Table 1. Clinical characterization of study cohort. Summary of demographic, clinical and genetic features of the cohort of subjects (PD and CTRL) whose purified CD14+ monocytes were used for integrated genomic analysis. [file 13024_2022_554_MOESM1_ESM.docx]

**Supplementary Table 1. Clinical characterization of study cohort.**

Summary of demographic, clinical and genetic features of the cohort of subjects (PD and CTRL) whose purified CD14+ monocytes were used for integrated genomic analysis.

|  | PD | PD/GBA | CTRL | CTRL/GBA |
| --- | --- | --- | --- | --- |
| Number of subjects | 56 | 23 | 66 | 13 |
| Gender (% of females) | 30% | 61% | 67% | 54% |
| Race (% of Northern European ancestry) | 100% | 100% | 100% | 100% |
| Age (mean, min-max) | 68.7 (47-88) | 60.3 (28-77) | 67 (37-86) | 58.3 (38-81) |
| % of N370 GBA-variant carriers | 0 | 74% | 0 | 46% |
| % of G2019S LRRK2-variants carriers | 7% | 4.3% | 1.5% | 0 |
