# Supplementary material for: Transcriptome deregulation of peripheral monocytes and whole blood in GBA-related Parkinson’s disease
Source: Mol Neurodegener. 2022 Aug 17;17:52. doi: 10.1186/s13024-022-00554-8 (PMC9386994; doi:10.1186/s13024-022-00554-8)
Supplement: Supplementary file 2 — Additional file 2: Supplementary Table 2. Targeted pathway enrichment in PD/GBA vs CTRL/GBA. List of endolysosomal pathways (from GO terms and curated pathways, i.e. ubiquitin pathway) reported in Fig. 5. p-value of enrichment as per Fisher exact test of each pathway within the set of up-regulated (UP) and down-regulated (DOWN) genes. Significant enriched pathways (at p-value < 0.15) are highlighted in red. [file 13024_2022_554_MOESM2_ESM.docx]

**Supplementary Table 2. Targeted pathway enrichment in PD/GBA vs CTRL/GBA.**

List of endolysosomal pathways (from GO terms and curated pathways, i.e. ubiquitin pathway) reported in Fig. 5. p-value of enrichment as per Fisher exact test of each pathway within the set of up-regulated (UP) and down-regulated (DOWN) genes. Significant enriched pathways (at p-value < 0.15) are highlighted in red.

|  | UP_pVal | genes UP | DOWN_pVal | genes DOWN |
| --- | --- | --- | --- | --- |
| NCL | 1 |  | 0.3809873 | TPP1 |
| endolysosome_GO:0036020 | 1 |  | 0.3471233 | AP2A1 |
| mucolipidosis_oligosaccaridosis | 1 |  | 0.3809873 | ﻿SLC17A5 |
| vacuolar_membrane_GO:0005774 | 1 |  | 0.52566 | ﻿VAC14 |
| vesicle_membrane_GO:0012506 | 1 |  | 0.03327179 | RIPOR1, ﻿SYNJ1, ﻿ATP2A2 |
| proteasome_genes | 1 |  | 0.6731331 | ﻿PSMB5 |
| Endocytic_vesicle_membrane_GO:0030666 | 0.2526243 | CAMK2G | 0.7863309 | AP2A1 |
| phagocitic_vescicle_membrane_GO:0030670 | 1 |  | 0.1067676 | RAPGEF1, ﻿TLR1, ﻿RAB11B, ﻿PIK3R4 |
| synaptic_vesicles_membrane_GO:0030672 | 1 |  | 0.2200207 | SLC17A5, RAB11 b |
| secretory_granules_GO:0030667 | 1 |  | 0.4707626 | COBP1, ﻿B4GALT1, ﻿C5AR1 |
| late_endosome_membrane_GO:0031902 | 1 |  | 0.9298442 | VAC14 |
| early_endosome_membrane_GO:0031901 | 1 |  | 0.8240028 | ﻿VAC14, ﻿LLGL1 |
| cytoplasmic_vescicles_membrane_GO:0030659 | 0.6527217 | GDE1 | 0.1029702 | ﻿ZDHHC8, ﻿ANKRD27, ﻿SNX9, ﻿MYOF, ﻿IFNGR2, ﻿AP1G1 |
| endosome_membrane_GO:0010008 | 1 |  | 0.7561683 | CLCN6, ﻿VAC14, ﻿ARHGAP1, ﻿PIP5K1C, ﻿PLEKHM1 |
| vesicle_mediated_transport_GO:0016192 | 0.3286001 | ﻿ARF5, ﻿CHML, ﻿HSPA1L | 0.5465935 | ﻿TSC2, ﻿CYTH1, ﻿COPB1, ﻿SEC16A, ﻿AP1G1, ﻿SEC24C, ﻿AP2A1 |
| lysosomal_membrane_GO:0005765 | 1 | ﻿CLCN6, ﻿ATP11A, ﻿SLC17A5, ﻿RPTOR, ﻿AP1G1, ﻿SZT2, ﻿PLEKHM1 | 0.6965521 |  |
| ubiquitin_genes | 0.5310891 | ﻿MIB1, ﻿ATG7, ﻿RING1 | 0.6525329 | ﻿MGRN1, ﻿HERC2, ﻿RNF144B, ﻿RNF185, ﻿RNF44, ﻿SYVN1, ﻿KMT2D, ﻿RNF26 |
| lysosomes_database | 0.3546838 |  | 0.6785794 | ﻿CLCN6, ﻿ATP11A, ﻿ANKRD27, ﻿SLC17A5, ﻿ANXA11, ﻿RPTOR, ﻿TPP1, ﻿AP1G1, ﻿ADA |
| Golgi_membrane_GO:0000139 | 0.6442496 | ﻿RER1, ﻿ABCG1, ﻿RAB33B, ﻿ENTPD6 | 0.3109093 | ﻿PKD1, ﻿TNFRSF1A, ﻿B4GALT1, ﻿VAC14, ﻿LFNG, ﻿RGP1, ﻿CYTH1, ﻿COPB1, ﻿LLGL1, ﻿SEC16A, ﻿NOTCH1, ﻿B4GALT5, ﻿IFNGR2, ﻿AP1G1﻿, ﻿AP1G1, ﻿SLC9A8,﻿SCAMP4 |
| ER_membrane_GO:0005789 | 0.2552859 | ﻿RETSAT, ﻿TRAM1, ﻿ALDH3A2, ﻿ABCC6, ﻿DAD1, ﻿FMO5, ﻿TMX1, ﻿CYB5R1, ﻿ABCG1 | 0.6093013 | ﻿PNPLA6, ﻿CYB5R3, ﻿DMPK, ﻿ERLIN1, ﻿COPB1, ﻿RNF185, ﻿KSR1, ﻿SEC16A, ﻿NOTCH1, ﻿IFNGR2, ﻿SYVN1, ﻿SELENON, ﻿RNF26, ﻿ATP2A2, ﻿CTDNEP1, ﻿SEC24C, ﻿POM121, ﻿TMEM189, ﻿POM121C |
