# Supplementary material for: Transcriptome deregulation of peripheral monocytes and whole blood in GBA-related Parkinson’s disease
Source: Mol Neurodegener. 2022 Aug 17;17:52. doi: 10.1186/s13024-022-00554-8 (PMC9386994; doi:10.1186/s13024-022-00554-8)
Supplement: Supplementary file 3 — Additional file 3: Supplementary Table 3. GO terms pathways enrichment analysis. The table reports the GO terms and ID for the pathways that were tested for enrichment analysis of differentially expressed genes between the CTRL/GBA and PD/GBA cohorts. [file 13024_2022_554_MOESM3_ESM.docx]

**Supplementary Table 3**. **GO terms pathways enrichment analysis**.

The table reports the GO terms and ID for the pathways that were tested for enrichment analysis of differentially expressed genes between the CTRL/GBA and PD/GBA cohorts.

| **GO term** | **GO ID** |
| --- | --- |
| vesicle-mediated transport | GO:0016192 |
| endolysosome | GO:0036020 |
| lysosomal membrane | GO:0005765 |
| vacuolar membrane | GO:0005774 |
| mitochondrial membrane | GO:0031966 |
| Golgi membrane | GO:0000139 |
| autophagosome membrane | GO:0000421 |
| vesicle membrane | GO:0012506 |
| endosome membrane | GO:0010008 |
| membrane invagination | GO:0010324 |
| membrane biogenesis | GO:0044091 |
| membrane assembly | GO:0071709 |
| membrane docking | GO:0022406 |
| phagocytic vesicles membrane | GO:0030670 |
| ER membrane | GO:0005789 |
| peroxisomal membrane transport | GO:0015919 |
| mitochondrial membrane fusion | GO:1990613 |
| synaptic vesicles membrane | GO:0030672 |
| secretory granule membrane | GO:0030667 |
| mitochondria outer membrane | GO:0005741 |
| mitochondrial inner membrane | GO:0005743 |
| coated vesicles membrane | GO:0030662 |
| endocytic vesicles membrane | GO:0030666 |
| cytoplasmic vesicles membrane | GO:0030659 |
| transport vesicles membrane | GO:0030658 |
| autophagosome membrane docking | GO:0016240 |
| peroxisome membrane biogenesis | GO:0016557 |
| exocytic vesicles membrane | GO:009950 |
| mitochondrial membrane fission | GO:0090149 |
| early endosome membrane | GO:003190 |
| late endosome membrane | GO:0031902 |
| early phagosome membrane | GO:0036186 |
| complement activation | GO:0006956 |
| extracellular exosomes: | GO:0070062 |
| exosomes (RNA complex) | GO:0000178 |
| extracellular exosomes complex | GO:1990563 |
| extracellular exosomes biogenesis | GO:0097734 |
| extracellular exosomes micropinocytosis | GO:0061707 |
| extracellular exosomes assembly | GO:0071971 |
| cytoplasmic exosomes | GO:0000177 |
| trans-synaptic signaling via exosomes | GO:0099157 |
| regulation of extracellular exosomes assembly | GO:1903551 |
| clathrin dependent exosomes assembly | GO:1990771 |
| positive regulator of exosome assembly | GO:1903553 |
| negative regulator of exosome assembly | GO:1903552 |
| RNA polymerase exosomes dependent | GO:0030847 |
| exosomal secretion | GO:1990182 |
| regulation of exosomal secretion | GO:1903541 |
| negative regulation exosomal secretion | GO:1903542 |
| positive regulation exosomal secretion | GO:1903543 |
