# Supplementary material for: Transcriptome deregulation of peripheral monocytes and whole blood in GBA-related Parkinson’s disease
Source: Mol Neurodegener. 2022 Aug 17;17:52. doi: 10.1186/s13024-022-00554-8 (PMC9386994; doi:10.1186/s13024-022-00554-8)
Supplement: Supplementary file 4 — Additional file 4: Supplementary Table 4. Number of subjects (PD and CTRL) with different GBA variants, reported in the text. [file 13024_2022_554_MOESM4_ESM.docx]

**Supplementary Table 4.** Number of subjects (PD and CTRL) with different *GBA* variants, reported in the text.

| ***GBA* variant** | **CTRL** | **PD** |
| --- | --- | --- |
| *84GG* | 2 | 1 |
| *84GG/T369M* | 0 | 1 |
| *E326K* | 3 | 1 |
| *L444P/A456P/RecNciI* | 0 | 1 |
| *LRRK2* | 1 | 4 |
| *N370S* | 5 | 15 |
| *N370S_LRRK2* | 0 | 1 |
| *N370S_N370S* | 0 | 1 |
| *N370S/RecNciI* | 1 | 0 |
| *none* | 65 | 52 |
| *R496H* | 1 | 1 |
| *V394L* | 1 | 1 |
|  |  |  |
