# Supplementary material for: Transcriptome deregulation of peripheral monocytes and whole blood in GBA-related Parkinson’s disease
Source: Mol Neurodegener. 2022 Aug 17;17:52. doi: 10.1186/s13024-022-00554-8 (PMC9386994; doi:10.1186/s13024-022-00554-8)
Supplement: Supplementary file 5 — Additional file 5: Supplementary Table 5. Summary of demographic, clinical and genetic features of the PPMI cohort (whole blood) of subjects (PD and CTRL) in this study. [file 13024_2022_554_MOESM5_ESM.docx]

**Supplementary Table 5.** Summary of demographic, clinical and genetic features of the PPMI cohort (whole blood) of subjects (PD and CTRL) in this study.

|  | PD | PD/GBA | CTRL | CTRL/GBA |
| --- | --- | --- | --- | --- |
| Number of subjects | 616 | 127 | 362 | 165 |
| Gender (% of females) | 42% | 44% | 46% | 66% |
| Race (% of white) | 91% | 97% | 91% | 97% |
| Age (mean, min-max) | 63.0 (32-90) | 62.7 (32- 90) | 57.8 (19-84) | 56.4 (25-86) |
