# Supplementary material for: Transcriptome deregulation of peripheral monocytes and whole blood in GBA-related Parkinson’s disease
Source: Mol Neurodegener. 2022 Aug 17;17:52. doi: 10.1186/s13024-022-00554-8 (PMC9386994; doi:10.1186/s13024-022-00554-8)
Supplement: Supplementary file 6 — Additional file 6: Supplementary Table 6. Overlapping genes between monocytes and PPMI cohort (whole blood) in manifesting vs non-manifesting GBA-carriers. List of overlapping genes differentially expressed in monocytes and whole blood in manifesting vs non-manifesting carriers. Gene name, description of gene function and logFC and FDR in monocytes and whole blood are reported. In “grey” genes that represent PD-GWAS hits are highlighted. [file 13024_2022_554_MOESM6_ESM.docx]

**Supplementary Table 6. Overlapping genes between monocytes and PPMI cohort (whole blood) in manifesting vs non-manifesting GBA-carriers.**

List of overlapping genes differentially expressed in monocytes and whole blood in manifesting vs non-manifesting carriers. Gene name, description of gene function and logFC and FDR in monocytes and whole blood are reported. In “grey” genes that represent PD-GWAS hits are highlighted.

| **Gene** | **Description** | **logFC**  **(monocytes)** | **FDR**  **(monocytes)** | **logFC**  **(whole blood)** | **FDR**  **(whole blood)** |
| --- | --- | --- | --- | --- | --- |
| **ABCG1** | Phospholipids and cholesterol cellular transport | 0.961465911314694 | 0.0459624608087888 | 0.23968699380249 | 0.0401794415274729 |
| **AC007342.3** |  | 0.57210658223937 | 0.0116606709179357 | 0.283178419304563 | 0.00823687604203322 |
| **ADD2** | β adducin gene / assembly of the spectrin-actin network | -0.912023451806256 | 0.0483997995924918 | -0.141246049132182 | 0.0272036288413351 |
| **ARHGAP9** | Rho GTPase cycle, Neutrophil degranulation, hematopoietic cells binding to extracellular matrix | 0.268124153126283 | 0.0101875885983114 | 0.235030480304674 | 0.0182020701710991 |
| **GPI** | Glucose-6-Phosphate Isomerase / lipid anchor for cell-surface proteins | -0.186214271347422 | 0.0400440200555539 | -0.143507177670195 | 0.0244152975439078 |
| **ING4** | Inhibitor Of Growth Family Member 4 / tumor suppressor / apoptosis | 0.198598151130411 | 0.0464557273237816 | 0.0916280556322153 | 0.0191529717760595 |
| **MAP4** | Microtubule-associated protein 4 /microtubule associated protein related to TAU and STMN1 **(GWAS hit)** | -0.322611615992687 | 0.0170722305590492 | -0.149920509213461 | 0.0113155729337565 |
| **METRNL** | Meteorin-like/ expressed in activated macrophages | -0.376611923492884 | 0.0348741627170725 | 0.158803045181993 | 0.0354498086303043 |
| **MTHFD1** | methylenetetrahydrofolate dehydrogenase 1 | -0.293226296096193 | 0.0459624608087888 | -0.137985273190468 | 0.0464314181999015 |
| **QPCT** | Glutaminyl-peptide cyclotransferase / metabolism APP amyloid-beta peptides | 0.294663529079197 | 0.035839785633954 | -0.0017683378784259 | 0.98649984057878 |
| **RAB33B** | Ras-related protein Rab-33B / autophagosome formation / Golgi homeostasis and trafficking | 0.272912365448382 | 0.0236446424550435 | 0.141779591452484 | 0.0273850778831599 |
| **RGL2** | Ral guanine nucleotide dissociation stimulator-like 2 **(GWAS hit)** | 0.177237657505668 | 0.0348741627170725 | 0.177270448436024 | 0.0335312353454597 |
| **RSRP1** | Arginine And Serine Rich Protein 1 | 0.294899104257617 | 0.016281087770457 | 0.109410987627835 | 0.0267370496570794 |
| **SLC7A1** | High affinity cationic amino acid transporter 1 | -0.395917432544339 | 0.0363324920815811 | -0.255699529915818 | 0.0370438163669751 |
| **SMAD3** | TGF-beta signaling pathway | -0.456474255965785 | 0.0156414130224551 | -0.168543588997348 | 0.0169091684342028 |
| **SUPV3L1** | ATP-dependent RNA helicase SUPV3L1, mitochondrial/ maintenance of mtDNA and chromatine | -0.332545474992509 | 0.0101676754748312 | -0.0859904726039504 | 0.0172965956631605 |
