# Supplementary material for: Transcriptome deregulation of peripheral monocytes and whole blood in GBA-related Parkinson’s disease
Source: Mol Neurodegener. 2022 Aug 17;17:52. doi: 10.1186/s13024-022-00554-8 (PMC9386994; doi:10.1186/s13024-022-00554-8)
Supplement: Supplementary file 7 — Additional file 7: Supplementary Table 7. Up-regulated and down-regulated pathway related to vesicle and membrane trafficking in isolated CD14+ monocytes and whole blood. The table shows the list of pathways that were found to be deregulated in both isolated CD14+ monocytes and whole blood in the PD/GBA vs CTRL/GBA groups (top), the deregulated genes in each pathway (first column), and the directionality of dysregulation (second and third column). Red “x” indicates genes dysregulated in whole blood, black “x” represents genes dysregulated in isolated CD14+ monocytes. [file 13024_2022_554_MOESM7_ESM.docx]

**Supplementary Table 7. Up-regulated and down-regulated pathway related to vesicle and membrane trafficking in isolated CD14+ monocytes and whole blood.** The table shows the list of pathways that were found to be deregulated in both isolated CD14+ monocytes and whole blood in the PD/GBA vs CTRL/GBA groups (top), the deregulated genes in each pathway (first column), and the directionality of dysregulation (second and third column). Red “x” indicates genes dysregulated in whole blood, black “x” represents genes dysregulated in isolated CD14+ monocytes.

|  | Monocytes_GBA/PD VS GBA/CTRL | Whole Blood_ GBA/PD VS GBA/CTRL | GOCC_SECRETORY_GRANULE | GOBP_VESICLE_MEDIATED-TRANSPORT | GOCC_SECRETORY_VESICLES | GOCC_VESICLE_LUMEN | GOCC_TERTIARY_GRANULES | GOCC_VACUOLES | GOCC_ENDOSOME | GOCC_TERTIARY_GRANULE_LUMEN |
| --- | --- | --- | --- | --- | --- | --- | --- | --- | --- | --- |
| AAK1 | DOWN |  |  | x |  |  |  |  |  |  |
| ABCG1 |  | UP |  |  |  |  |  |  | x |  |
| ADGRG3 |  | UP | x |  | x |  |  |  |  |  |
| ANKRD13D |  | UP |  |  |  |  |  |  | x |  |
| ANKRD27 | DOWN |  |  | x |  |  |  |  |  |  |
| ANXA11 | DOWN |  |  | x |  |  |  |  |  |  |
| AP1G1 | DOWN |  |  | x |  |  |  |  |  |  |
| AP2A1 | DOWN |  |  | x |  |  |  |  |  |  |
| ARHGAP1 | DOWN |  |  | x |  |  |  |  |  |  |
| ARHGAP9 | UP |  | x |  | x |  |  |  |  |  |
| ARHGAP9 |  | UP | x |  | x | x |  |  |  |  |
| ARRDC3 |  | UP |  |  |  |  |  | x | x |  |
| ATG7 | UP |  | x |  | x |  |  |  |  |  |
| ATP2A2 | DOWN |  |  | x |  |  |  |  |  |  |
| BIN3 | DOWN |  |  | x |  |  |  |  |  |  |
| BST1 | UP |  | x |  | x |  |  |  |  |  |
| BTBD9 | DOWN |  |  | x |  |  |  |  |  |  |
| CA4 |  | UP | x |  | x |  |  |  |  |  |
| CAB39 |  | UP | x |  | x | x |  |  |  |  |
| CCDC189 |  | UP | x |  | x |  |  |  |  |  |
| CCR1 | DOWN |  |  | x |  |  |  |  |  |  |
| CD81 | DOWN |  |  | x |  |  |  |  |  |  |
| CDA |  | UP | x |  | x | x | x |  |  | x |
| CDK16 | DOWN |  |  | x |  |  |  |  |  |  |
| CDKN1B |  | UP |  |  |  |  |  |  | x |  |
| CEACAM3 |  | UP | x |  | x |  |  |  |  |  |
| CHI3L1 |  | UP | x |  | x | x |  |  |  |  |
| CHMP1B |  | UP |  |  |  |  |  | x | x |  |
| COPB1 | DOWN |  |  | x |  |  |  |  |  |  |
| CSF3R |  | UP |  |  |  |  |  | x |  |  |
| CSK | DOWN |  |  | x |  |  |  |  |  |  |
| CTNNB1 | DOWN |  |  | x |  |  |  |  |  |  |
| CXCL1 |  | UP | x |  | x | x | x |  |  | x |
| CYB5R1 | UP |  | x |  | x |  |  |  |  |  |
| CYTH1 | DOWN |  |  | x |  |  |  |  |  |  |
| DDIT3 |  | UP |  |  |  |  |  |  | x |  |
| DENND10 |  | UP |  |  |  |  |  |  | x |  |
| DNAJC3 |  | UP | x |  | x | x |  | x |  |  |
| DOK3 |  | UP | x |  | x |  | x |  |  |  |
| DPYSL2 | DOWN |  |  | x |  |  |  |  |  |  |
| EPN1 | DOWN |  |  | x |  |  |  |  |  |  |
| EXOC8 |  | UP |  |  |  |  |  |  | x |  |
| FCAR |  | UP | x |  | x |  | x |  |  |  |
| FGL2 | UP |  | x |  | x |  |  |  |  |  |
| FLNA | DOWN |  |  | x |  |  |  |  |  |  |
| FOLR3 |  | UP | x |  | x | x | x |  |  | x |
| FPR2 | DOWN |  |  | x |  |  |  |  |  |  |
| FUCA2 | UP |  | x |  | x |  |  |  |  |  |
| GABARAPL1 |  | UP |  |  |  |  |  | x |  |  |
| GIT1 | DOWN |  |  | x |  |  |  |  |  |  |
| GLIPR1 | UP |  | x |  | x |  |  |  |  |  |
| HEBP2 |  | UP | x |  | x | x |  | x |  |  |
| HEXB | UP |  | x |  | x |  |  |  |  |  |
| HK3 |  | UP | x |  | x | x |  |  |  |  |
| ITGAL | DOWN |  |  | x |  |  |  |  |  |  |
| LAMTOR4 |  | UP |  |  |  |  |  | x | x |  |
| LLGL1 | DOWN |  |  | x |  |  |  |  |  |  |
| LY96 |  | UP |  |  |  |  |  |  | x |  |
| MAP1LC3A |  | UP |  |  |  |  |  | x | x |  |
| MGRN1 | DOWN |  |  | x |  |  |  |  |  |  |
| MIB2 |  | UP |  |  |  |  |  |  | x |  |
| MNDA | UP |  | x |  | x |  |  |  |  |  |
| MPZ |  | UP |  |  |  |  |  | x |  |  |
| MYO1G | DOWN |  |  | x |  |  |  |  |  |  |
| NHLRC3 | UP |  | x |  | x |  |  |  |  |  |
| NOTCH1 | DOWN |  |  | x |  |  |  |  |  |  |
| NRBF2 |  | UP |  |  |  |  |  | x |  |  |
| PDPK1 | DOWN |  |  | x |  |  |  |  |  |  |
| PGLYRP1 |  | UP | x |  | x | x | x |  |  | x |
| PIK3R4 | DOWN |  |  | x |  |  |  |  |  |  |
| PIP4P2 |  | UP |  |  |  |  |  | x | x |  |
| PIP5K1C | DOWN |  |  | x |  |  |  |  |  |  |
| PLEKHM1 | DOWN |  |  | x |  |  |  |  |  |  |
| PRAM1 | DOWN |  |  | x |  |  |  |  |  |  |
| PTPN23 | DOWN |  |  | x |  |  |  |  |  |  |
| QPCT | UP |  | x |  | x |  |  |  |  |  |
| QPCT |  | UP | x |  | x | x | x |  |  | x |
| RAB11B | DOWN |  |  | x |  |  |  |  |  |  |
| RAB33B |  | UP |  |  |  |  |  |  | x |  |
| RABEPK |  | UP |  |  |  |  |  |  | x |  |
| RARA | DOWN |  |  | x |  |  |  |  |  |  |
| RGP1 | DOWN |  |  | x |  |  |  |  |  |  |
| RIN3 | DOWN |  |  | x |  |  |  |  |  |  |
| RRAGD |  | UP |  |  |  |  |  | x |  |  |
| RUBCNL |  | UP |  |  |  |  |  | x |  |  |
| S100A8 |  | UP | x |  | x | x |  |  |  |  |
| S100A9 |  | UP | x |  | x | x |  |  |  |  |
| SEC16A | DOWN |  |  | x |  |  |  |  |  |  |
| SEC24C | DOWN |  |  | x |  |  |  |  |  |  |
| SLC11A1 |  | UP | x |  | x |  | x | x | x |  |
| SLC15A3 |  | UP |  |  |  |  |  | x | x |  |
| SLC2A3 |  | UP | x |  | x |  | x |  |  |  |
| SLIRP | UP |  | x |  | x |  |  |  |  |  |
| SNX9 | DOWN |  |  | x |  |  |  |  |  |  |
| SPI1 | DOWN |  |  | x |  |  |  |  |  |  |
| SRC | DOWN |  |  | x |  |  |  |  |  |  |
| STX10 |  | UP |  |  | x |  |  |  |  |  |
| SYNJ1 | DOWN |  |  | x |  |  |  |  |  |  |
| SYTL3 | UP |  |  |  | x |  |  |  |  |  |
| TAMALIN | DOWN |  |  | x |  |  |  |  |  |  |
| TBC1D10C |  | UP | x |  | x |  | x |  |  |  |
| THBD |  | UP |  |  |  |  |  | x |  |  |
| TLR9 |  | UP |  |  |  |  |  | x | x |  |
| TM6SF1 |  | UP |  |  |  |  |  | x |  |  |
| TRAPPC14 |  | UP |  |  |  |  |  |  | x |  |
| TSC2 | DOWN |  |  | x |  |  |  |  |  |  |
| WASF2 | DOWN |  |  | x |  |  |  |  |  |  |
