# Supplementary material for: Transcriptome deregulation of peripheral monocytes and whole blood in GBA-related Parkinson’s disease
Source: Mol Neurodegener. 2022 Aug 17;17:52. doi: 10.1186/s13024-022-00554-8 (PMC9386994; doi:10.1186/s13024-022-00554-8)
Supplement: Supplementary file 8 — Additional file 8: Supplementary Fig. 1. Characterization of genetic background of donor population. a) Representation of PCA analysis of ancestry of MDS values from the cohort of 158 subjects (PD/GBA, PD, CTRL/GBA, CTRL) compared to 1000 Genome Project samples (Phase 3). The different ancestry are represented in distinct colors (Orange: African; Gold: Ad Mixed American; Green: East Asian; Blue: European; Purple: South Asian; Black: study cohort). B) PCA considering only overlap of MDS values of donor cohort (black) with European ancestry (blue) and AJ ancestry (light blue). Supplementary Figure 2. Normalization and quality control of RNA-seq data from isolated CD14+ monocytes. a) Violin plot representing the contribution of each of the surrogate variables (as explained in the text) to the variability of expression data of the study cohort and residual (158 subjects). b) Violin plot representing the contribution of technical, demographical and clinical variables to the variability of expression data of the study cohort and residual (158 subjects). c) Heatmap representing the results of linear regression between the surrogate variables utilized for data normalization and technical variables (from RNA-seq analysis) and metadata. Coefficient of linear regression is reported in the heatmap for each correlation pair. d) Distribution of MDS values of study cohort identified a clear clustering based on batches used for RNA-seq analysis (batches 1 to 4). e) After regression of SVs, variability of MDS values is significantly reduced, with no significant outliers and no clustering based on experimental batches. Supplementary Fig. 3. Differential expression of GBA in CD14+ isolated monocytes. Box plot representing differential expression levels (normalized expression count) of GBA in isolated CD14+ monocytes (a and b). In b) data from isolated CD14+ monocytes of GBA-carriers and non-carriers within PD and CTRL subjects were combined and compared. Each dot represents a subject. [file 13024_2022_554_MOESM8_ESM.docx]

**Transcriptome deregulation of peripheral monocytes and whole blood in *GBA*-related Parkinson’s disease**

Giulietta Maria Riboldi^1^, Ricardo A Vialle^2,3,4,5^, Elisa Navarro^2,3,4,5^, Evan Udine^2,3,4,5^, Katia de Paiva Lopes^2,3,4,5^, Amanda Allan^2,3,4,5^, Madison Parks^2,3,4,5^, Brooklyn Henderson^1^, Kelly Astudillo^1^, Charalambos Argyrou^2,3,4,5^, Maojuan Zhuang^2,3,4,5^, Tamjeed Sikder^2,3,7,8^, Oriol Narcis J.^2,3,4,5^, Shilpa Dilip Kumar^6^, William Janssen^6^, Allison Sowa^6^, Giacomo P Comi^9,10^, Alessio Di Fonzo^9,10^, John F. Crary^2,3,7,8^, Steven J Frucht^1^, Towfique Raj^2,3,4,5^

^1^ The Marlene and Paolo Fresco Institute for Parkinson's Disease and Movement Disorders, New York University Langone Health, 222 East 41st street, New York, NY 10017, United States of America

^2^ Nash Family Department of Neuroscience & Friedman Brain Institute, Icahn School of Medicine at Mount Sinai, [One Gustave L. Levy Place, New York, NY 10029](https://maps.google.com/?q=%20One%20Gustave%20L.%20Levy%20Place%20New%20York%20NY%2010029), United States of America.

^3^ Ronald M. Loeb Center for Alzheimer’s disease, Icahn School of Medicine at Mount Sinai, [One Gustave L. Levy Place, New York, NY 10029](https://maps.google.com/?q=%20One%20Gustave%20L.%20Levy%20Place%20New%20York%20NY%2010029), United States of America.

^4^ Department of Genetics and Genomic Sciences & Icahn Institute for Data Science and Genomic Technology, Icahn School of Medicine at Mount Sinai, [One Gustave L. Levy Place, Box 1498, New York, NY 10029,](https://maps.google.com/?q=One%20Gustave%20L.%20Levy%20Place%20New%20York%20NY%2010029) United States of America

^5^ Estelle and Daniel Maggin Department of Neurology, Icahn School of Medicine at Mount Sinai, [One Gustave L. Levy Place, Box 1137, New York, NY 10029,](https://maps.google.com/?q=One%20Gustave%20L.%20Levy%20Place%20New%20York%20NY%2010029) United States of America.

^6^ Microscopy Core and Advanced Bioimaging Center at the Icahn School of Medicine at Mount Sinai Center, [1468 Madison Avenue, Room 18-250, New York, NY 10029,](https://maps.google.com/?q=1468%20Madison%20Avenue%20New%20York%20NY%2010029) United States of America.

^7^ Department of Pathology, Icahn School of Medicine at Mount Sinai, [1468 Madison Avenue, Annenberg Building, 15th Floor, New York, NY 10029,](https://maps.google.com/?q=1468%20Madison%20Avenue%20New%20York%20NY%2010029) United States of America

^8^ Neuropathology Brain Bank & Research CoRE, Icahn School of Medicine at Mount Sinai, [1425 Madison Avenue, Room 9-22, New York, NY 10029,](https://maps.google.com/?q=1425%20Madison%20Avenue%20New%20York%20NY%2010029) United States of America

^9^ IRCCS Ca' Granda Ospedale Maggiore Policlinico, Neurology Unit, Via Francesco Sforza, 35, 20122 Milano MI, Italy

^10^Dino Ferrari Center, Neuroscience Section, Department of Pathophysiology and Transplantation, University of Milan, Via Francesco Sforza, 35, 20122 Milano MI, Italy

**Supplementary Figures**


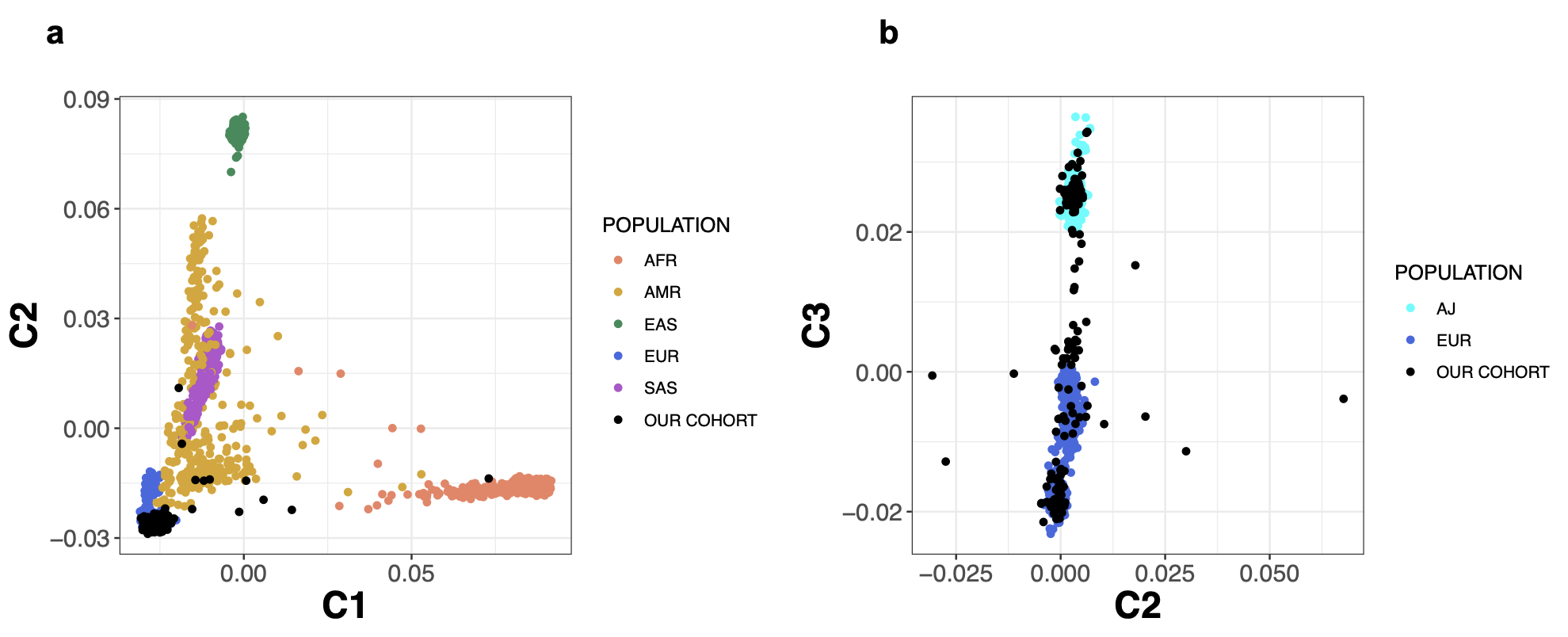


**Supplementary Fig. 1. Characterization of genetic background of donor population.**

a) Representation of PCA analysis of ancestry of MDS values from the cohort of 158 subjects (PD/GBA, PD, CTRL/GBA, CTRL) compared to 1000 Genome Project samples (Phase 3). The different ancestry are represented in distinct colors (Orange: African; Gold: Ad Mixed American; Green: East Asian; Blue: European; Purple: South Asian; Black: study cohort). B) PCA considering only overlap of MDS values of donor cohort (black) with European ancestry (blue) and AJ ancestry (light blue).


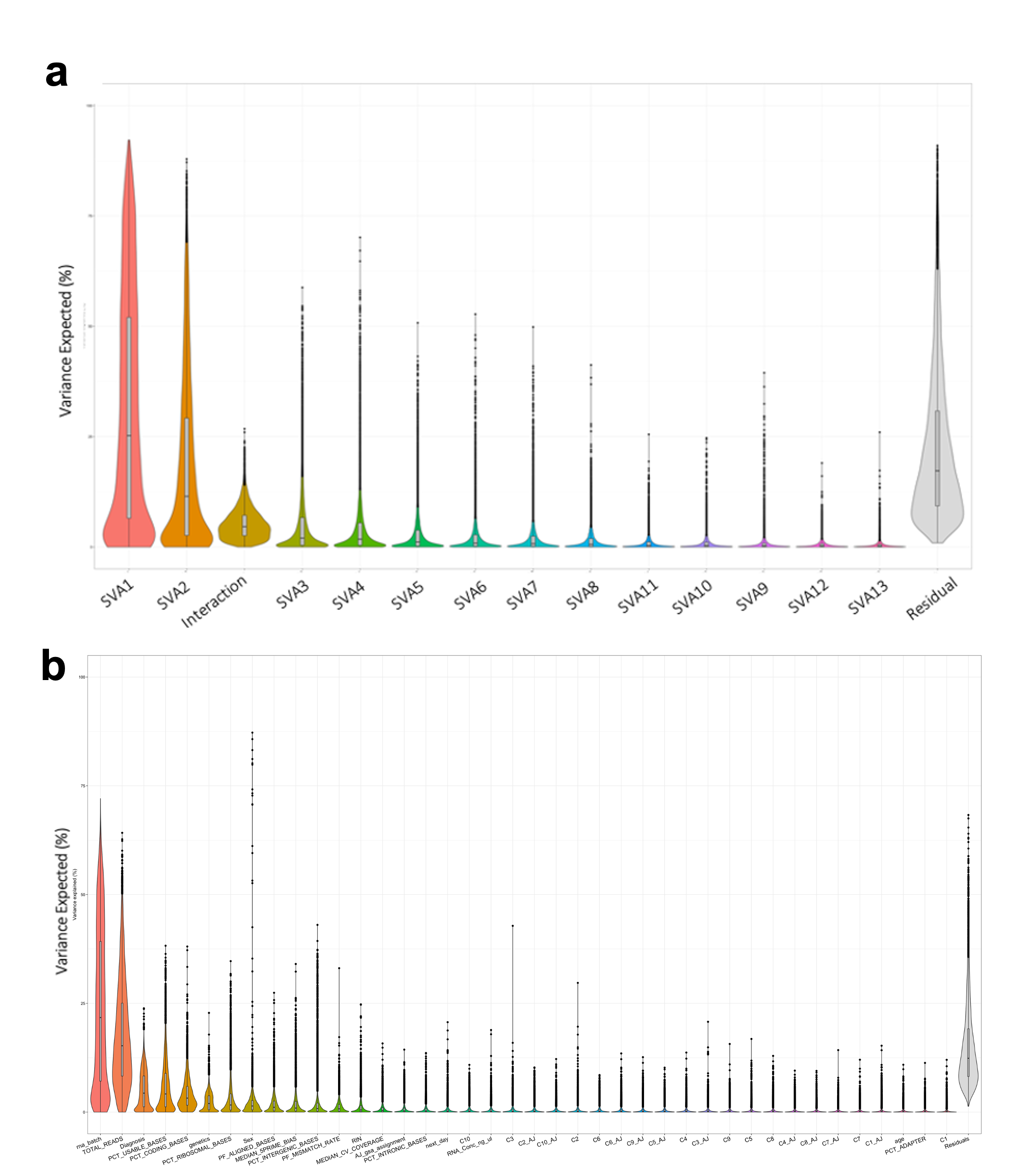


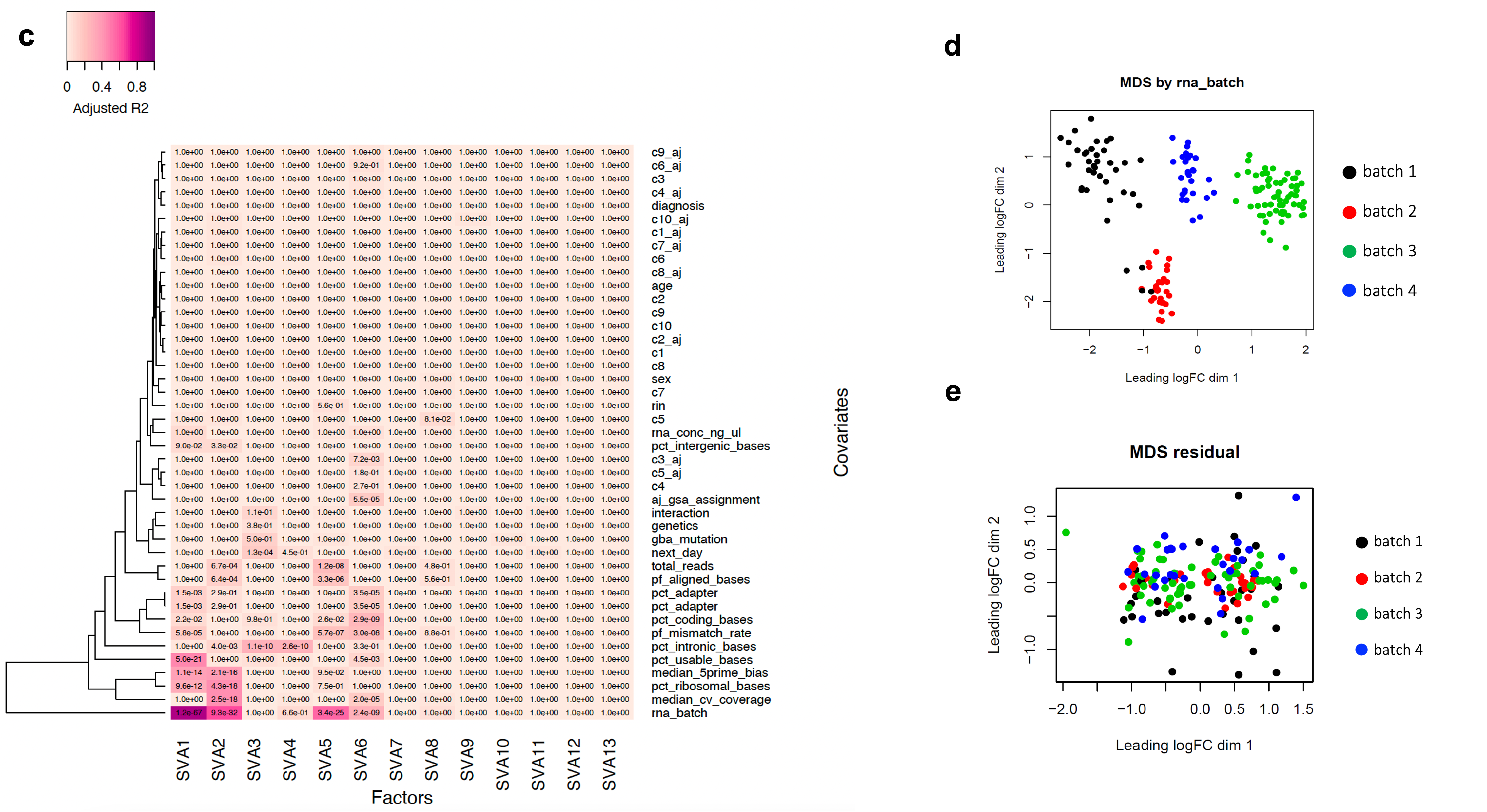


**Supplementary Fig. 2. Normalization and quality control of RNA-seq data from isolated CD14+ monocytes.**

a) Violin plot representing the contribution of each of the surrogate variables (as explained in the text) to the variability of expression data of the study cohort and residual (158 subjects).

b) Violin plot representing the contribution of technical, demographical and clinical variables to the variability of expression data of the study cohort and residual (158 subjects).

c) Heatmap representing the results of linear regression between the surrogate variables utilized for data normalization and technical variables (from RNA-seq analysis) and metadata. Coefficient of linear regression is reported in the heatmap for each correlation pair.

d) Distribution of MDS values of study cohort identified a clear clustering based on batches used for RNA-seq analysis (batches 1 to 4).

e) After regression of SVs, variability of MDS values is significantly reduced, with no significant outliers and no clustering based on experimental batches.

**
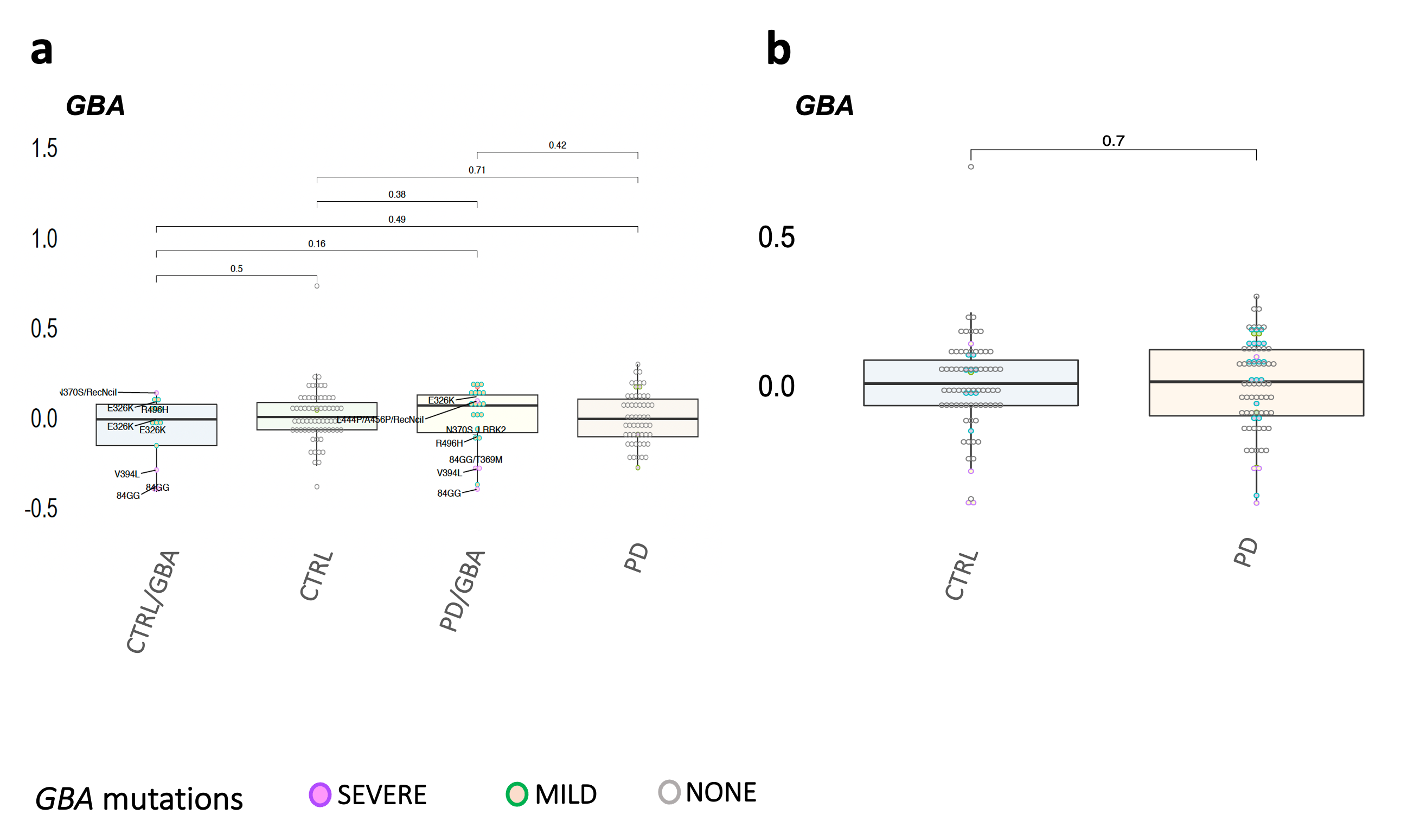
**

**Supplementary Fig. 3. Differential expression of *GBA* in CD14+ isolated monocytes.**

Box plot representing differential expression levels (normalized expression count) of *GBA* in isolated CD14+ monocytes (a and b). In b) data from isolated CD14+ monocytes of *GBA*-carriers and non-carriers within PD and CTRL subjects were combined and compared. Each dot represents a subject. Dots are colored based on *GBA* mutations (as reported in the legend: *GBA* mild mutations (N370S, E326K, R496H), *GBA* severe mutations (L444P/A456P/RecNciI, V394L, 84GG, 84GG/T369M, N370S/RecNciI)). p-value of different expression levels is reported on top (statistics: Mann-Whitney U test). *GBA* variants different from N370S are labeled in the boxplot.

**
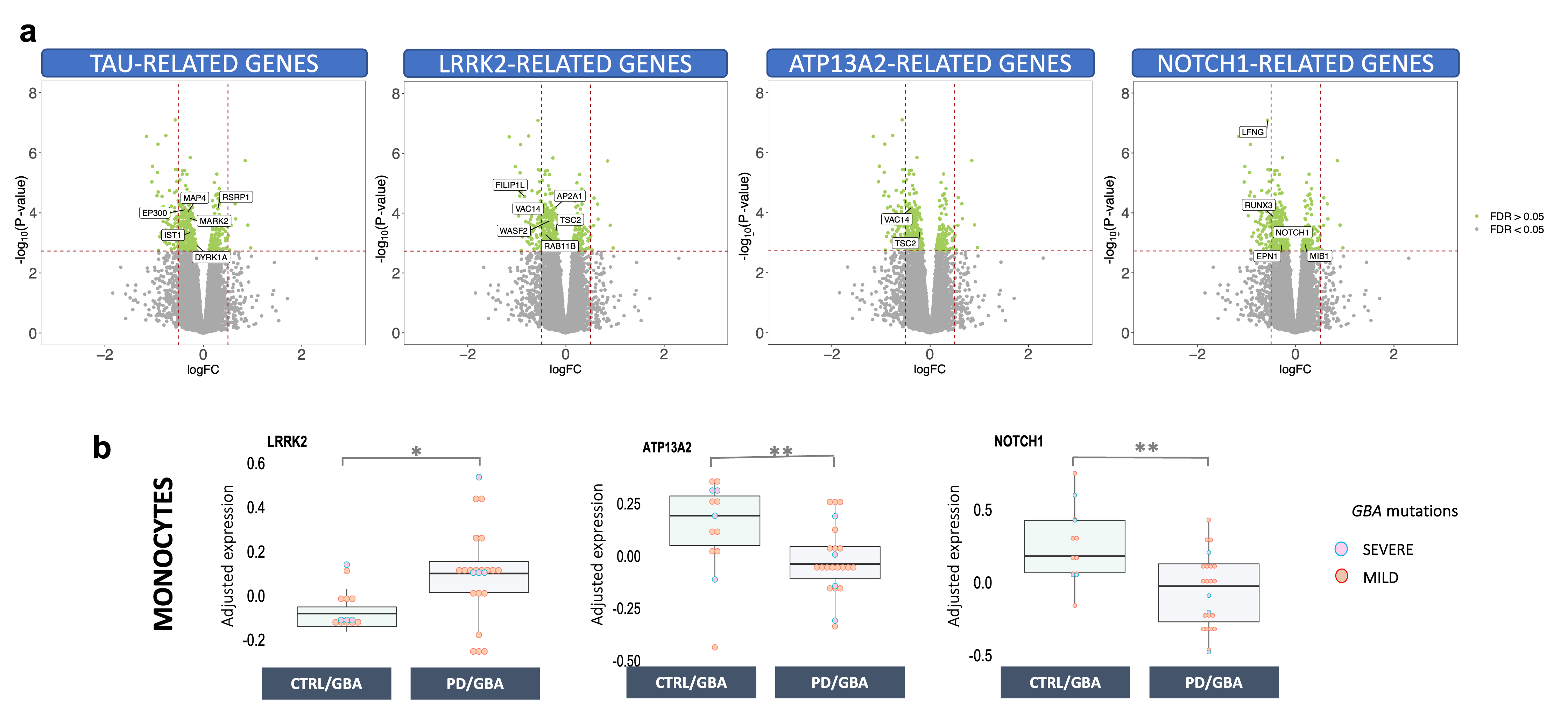
**

**Supplementary Fig. 4. Differential expression of target genes in manifesting and non-manifesting carriers in monocytes.**

a) Volcano plot showing differentially expressed genes with FDR < 0.05 (green dots) in isolated monocytes from manifesting vs non-manifesting carriers. Genes related to targeted pathways (*LRRK2*, *ATP13A2*, *NOTCH1* and *TAU*) are highlighted.

b) Box plots of differential levels of expression of the targeted genes (*ATP13A2*, *LRRK2*, *NOTCH1*), between manifesting and non-manifesting carriers in isolated monocytes. Each dot represents a subject. Dots are colored based on *GBA* mutations (as reported in the legend: *GBA* mild mutations (N370S, E326K, R496H), *GBA* severe mutations (L444P/A456P/RecNciI, V394L, 84GG, 84GG/T369M, N370S/RecNciI)). Asterisks indicate significant p-value (* = p-value < 0.05, ** = p-value < 0.01, *** = p-value < 0.001, statistics: Mann-Whitney U test).

**
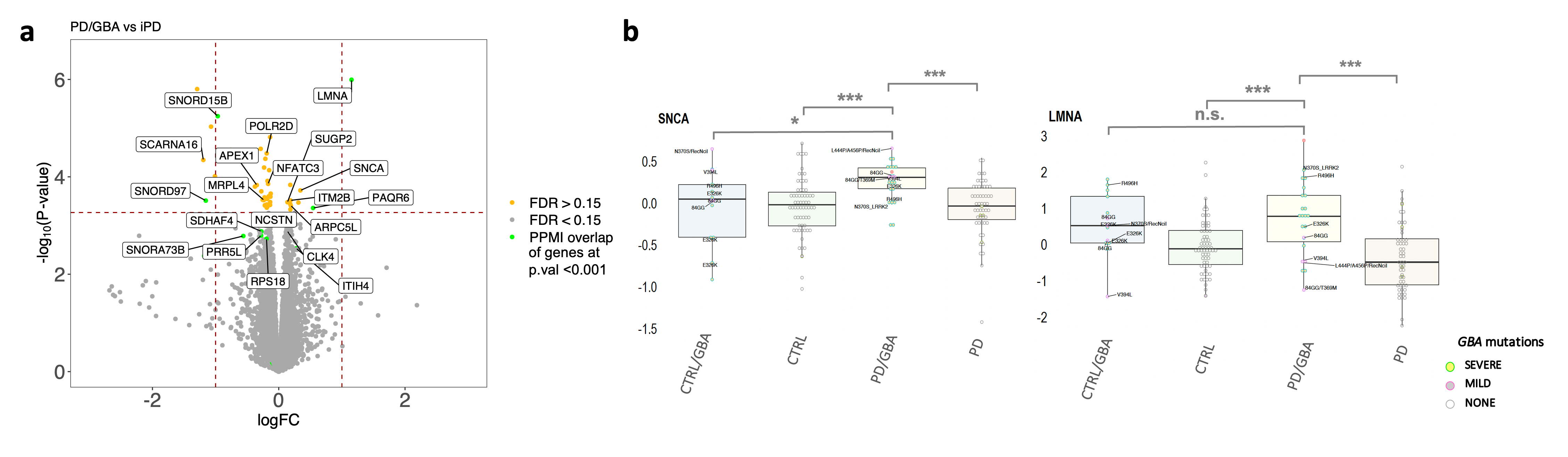
**

**Supplementary Fig. 5. Differential expression analysis in monocytes in PD patients with and without *GBA*-**v**ariants.**

a) Volcano-plot representing logFC (x-axes) and p-value (y-axes, -log_10_ p-value) of differential expressed genes between PD/GBA and PD as per nested interaction model. Highlighted in yellow are genes with FDR < 0.15 (44 total genes). ID labels of functionally relevant genes and of genes differentially expressed in whole blood (PPMI overlap of genes at nominal p-value <0.001) are reported in the plot.

b) Differential normalized expression count of *SNCA* and *LMNA* between PD/GBA and PD, compared to CTRL/GBA and CTRL subjects in isolated CD 14+ monocytes. Asterisks indicate significant p-value (* = p-value < 0.05, ** = p-value < 0.01, *** = p-value < 0.001, statistics: Mann-Whitney U test). Disease and genetic status are reported on the x-axes. Each dot represents a subject. Dots are colored based on *GBA* mutations (as reported in the legend: *GBA* mild mutations (N370S, E326K, R496H), *GBA* severe mutations (L444P/A456P/RecNciI, V394L, 84GG, 84GG/T369M, N370S/RecNciI)). *GBA* variants different from N370S are labeled in the boxplot.

**
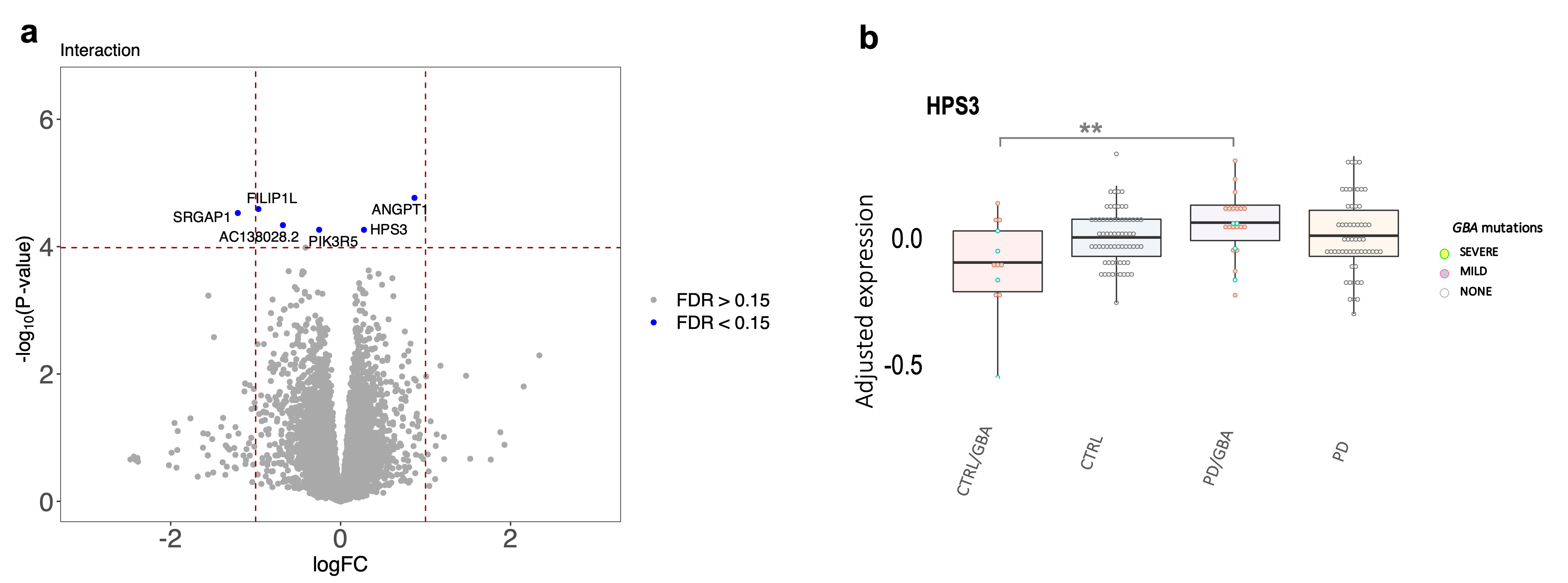
**

**Supplementary Fig. 6. Differential expression profiles across the four cohorts based on diagnosis and genetic status interaction.**

a) Volcano-plot representing log_2_ fold change (x-axes) and P-Value (y-axes, -log_10_ P-Value) of differential expressed genes based on diagnosis and genetics interaction between the four cohorts (PD/GBA, CTRL/GBA, PD, CTRL). Genes with FDR < 0.05 are highlighted in blue and labeled with their IDs. Differentially expressed genes encompassed: *ANGPT1* (angiopoietin gene involved in angiogenesis), *FILIP1L* (Filamin A Interacting Protein 1 Like), *AC138028.2* (novel transcript), *SRGAP1* (Slit-Robo GTPase-activating protein 1), *PIK3R5* (Phosphoinositide 3-kinase regulatory subunit 5, responsible for Ataxia with Oculomotor-Apraxia type 3), *HPS3* (Hermansky-Pudlak Syndrome 3 Protein, biogenesis of lysosomal organelle complex 2 subunit 1). b) Box plots representing expression levels (normalized expression count) of one differentially expressed targeted gene according to interaction term (diagnosis and genetics interaction). Disease and genetic status are labeled on the x-axes. Boxes are colored based on disease and genetic status. Each dot represents a subject. Dots are colored based on *GBA* mutations (as reported in the legend: *GBA* mild mutations (N370S, E326K, R496H), *GBA* severe mutations (L444P/A456P/RecNciI, V394L, 84GG, 84GG/T369M, N370S/RecNciI)). Asterisks indicate significant p-value (* = p-value < 0.05, ** = p-value < 0.01, *** = p-value < 0.001; statistics: Mann-Whitney U test).


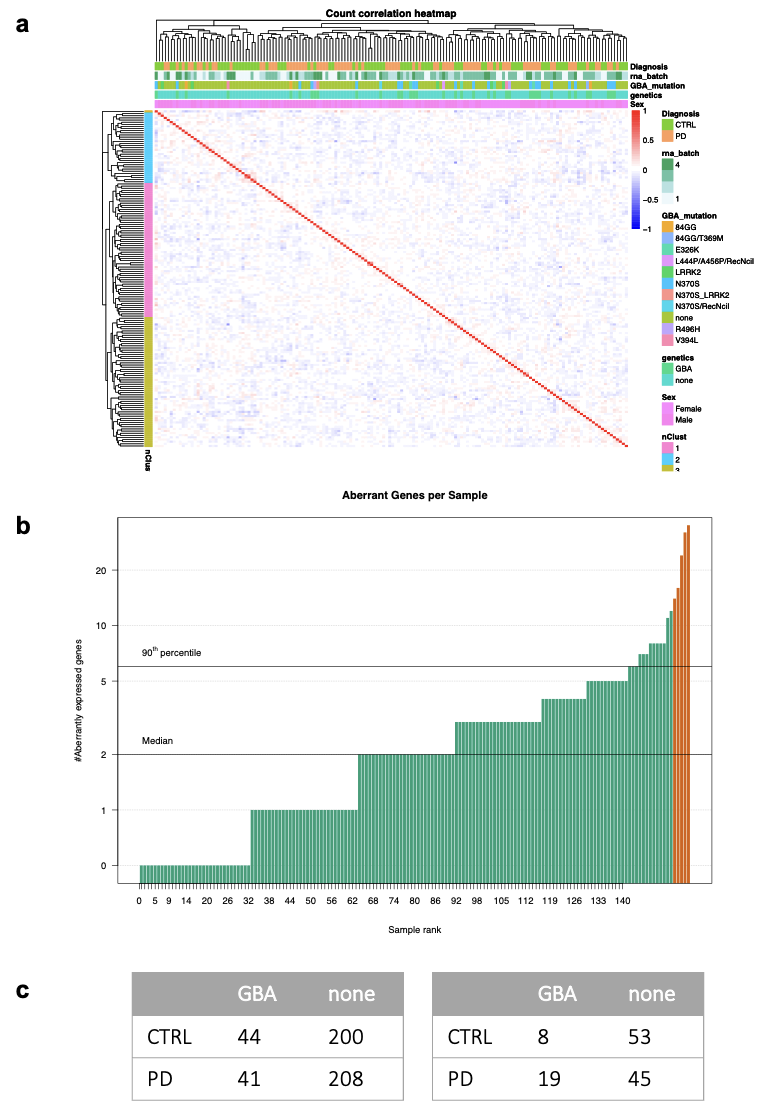


**Supplementary Fig. 7. QC for analysis of outlier genes.**

a) Normalization based on surrogate variables, as provided by the OUTRIDER script, of a total of 13711 genes (considering only genes with > 30% of total expression). Discrete relevant variables (Diagnosis, batches of RNAseq analysis (rna_batch), GBA mutations and GBA-related genetic status (carriers or non-carriers of GBA mutations), gender (Sex: male (M) and female (F)) are labeled at the top of the heatmap per each subject.

b) Bar-plot reporting number of outlier genes per each subject (out of 158 subjects). Highlighted in orange samples with outlier gene count above 0.1%.

c) Summary tables: on the left: number of outliers genes per cohort (PD/GBA, CTRL/GBA, PD, CTRL) (493 pairs); on the right: number of subjects per each cohort with at least one outlier gene (125 unique subjects total with at least one outlier gene).

**
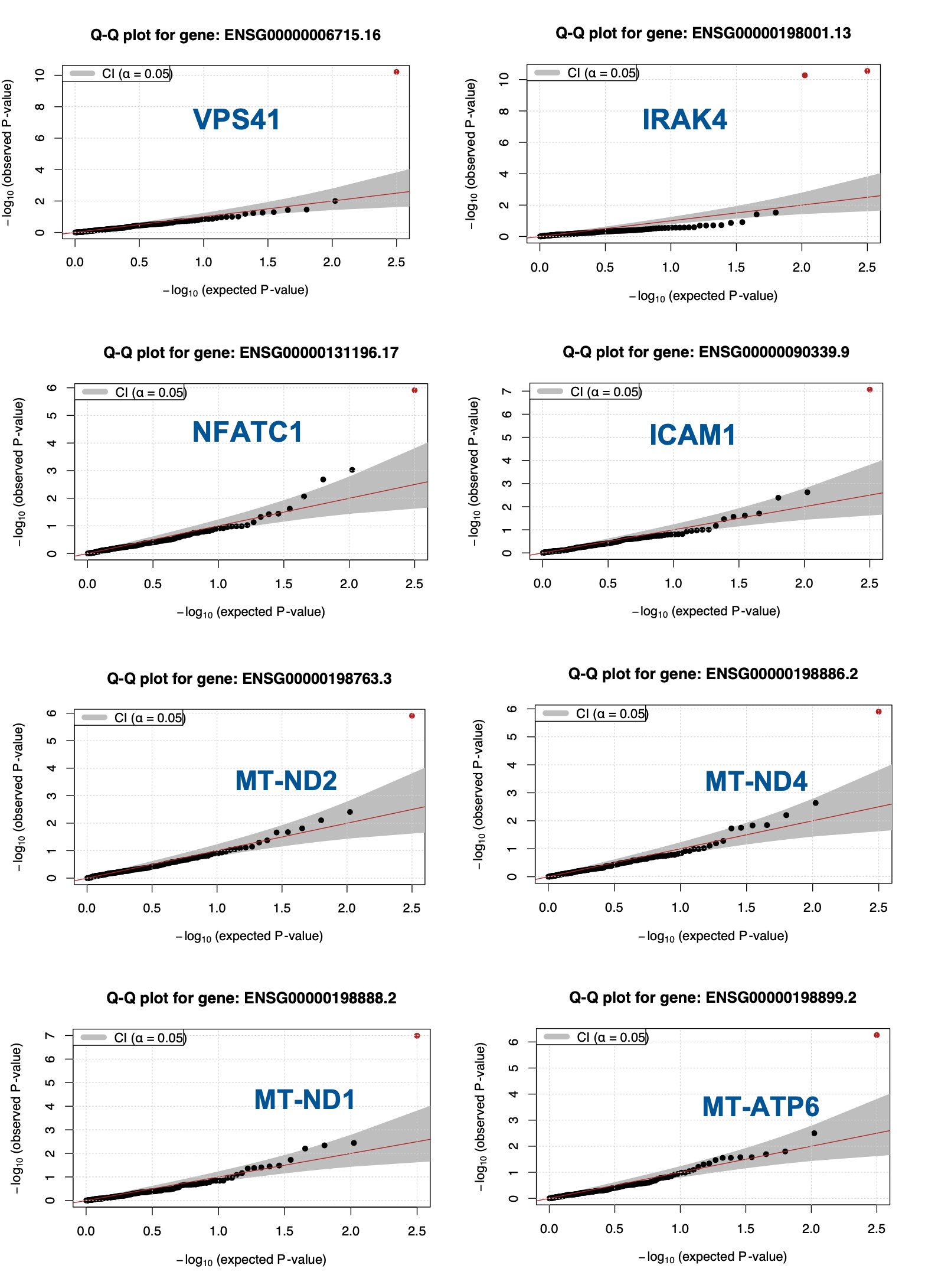
**

**Supplementary Fig. 8. Scatter plot of selected genes identified through the OUTRIDER method.**

Scatter plot representing -log_10_ (p-value) of  outliers genes identified in previous analysis.

**
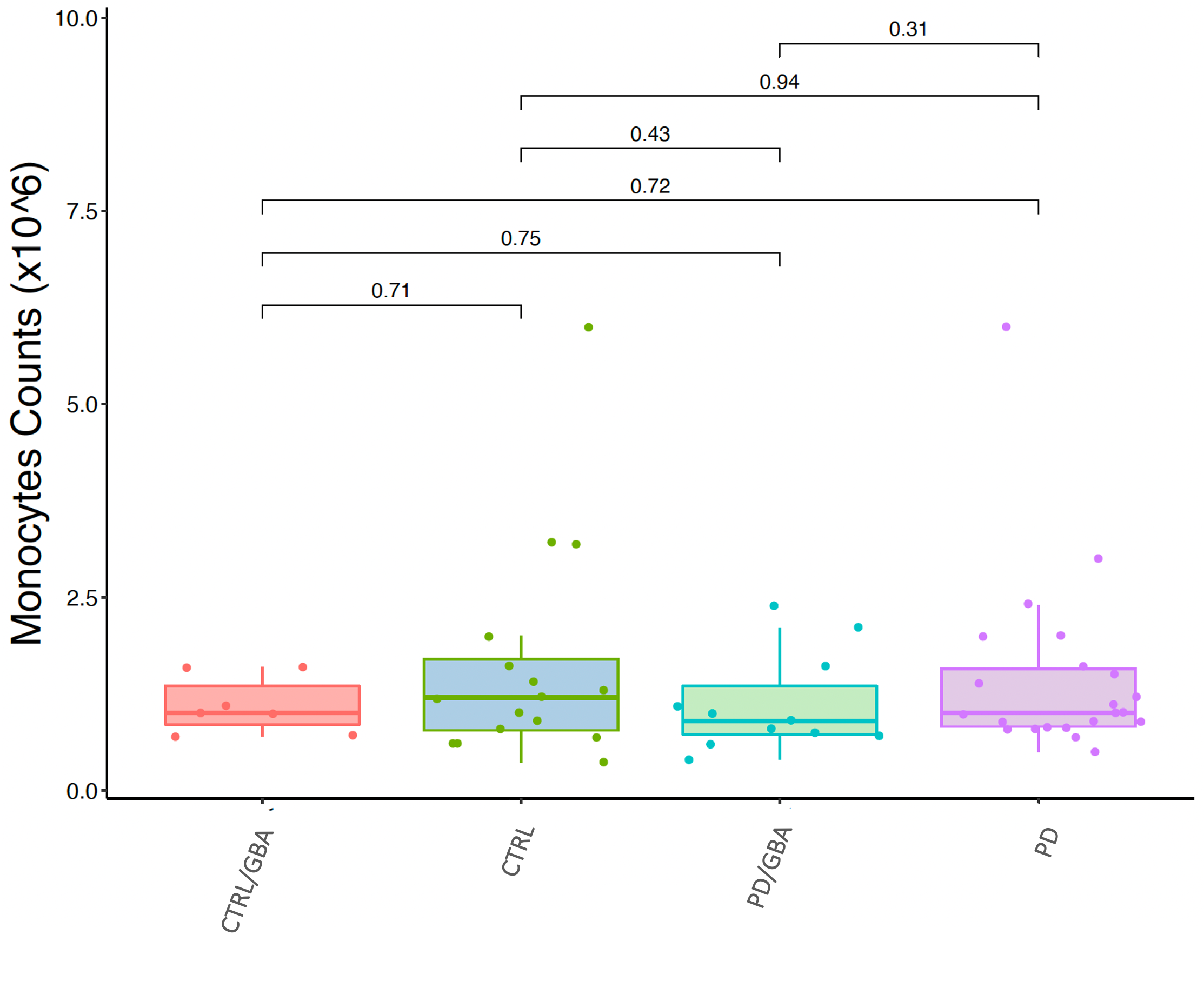
**

**a**

**
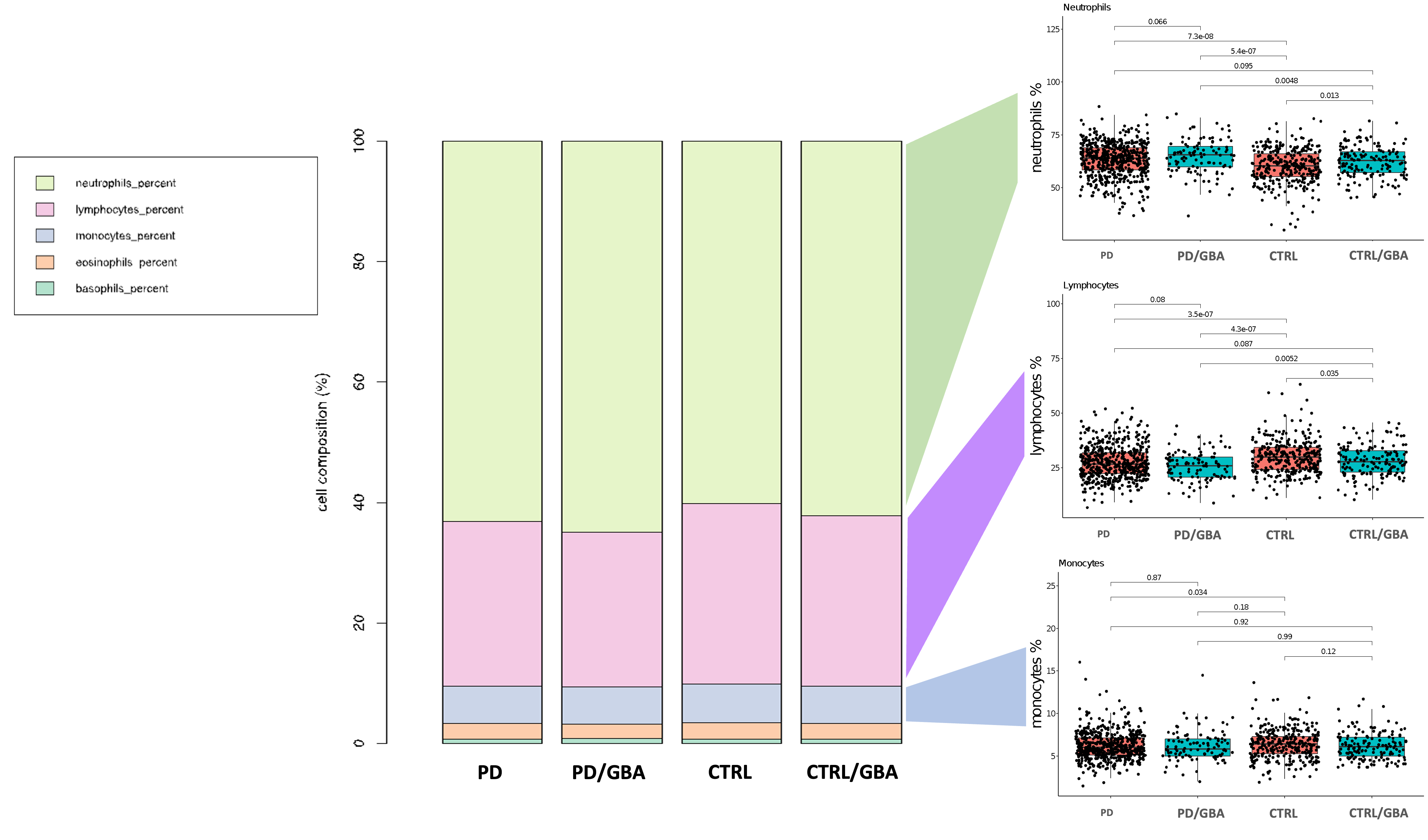
**

**b**

**Supplementary Fig. 9. Cell proportion across patient and control cohorts.**

a) Monocyte counts (x10^6^) in the GBA/PD, PD, GBA/CTRL, and CTRL groups in the cohort of isolated monocytes (NYMD cohort). Each dot represents the cell count of one subject. Cell count was available for 57 subjects in our cohort. Statistical analysis according to the Wilcoxon test is reported for the comparison between the four groups.

b) Cell proportion (neutrophils, monocytes, lymphocytes, eosinophils, basophils) within whole blood samples in the four groups (GBA/PD, PD, GBA/CTRL, and CTRL) in the PPMI cohort. Comparison of each cell type percent across the four groups is reported on the box plots on the right side of the figure (statistical analysis: Wilcoxon test).


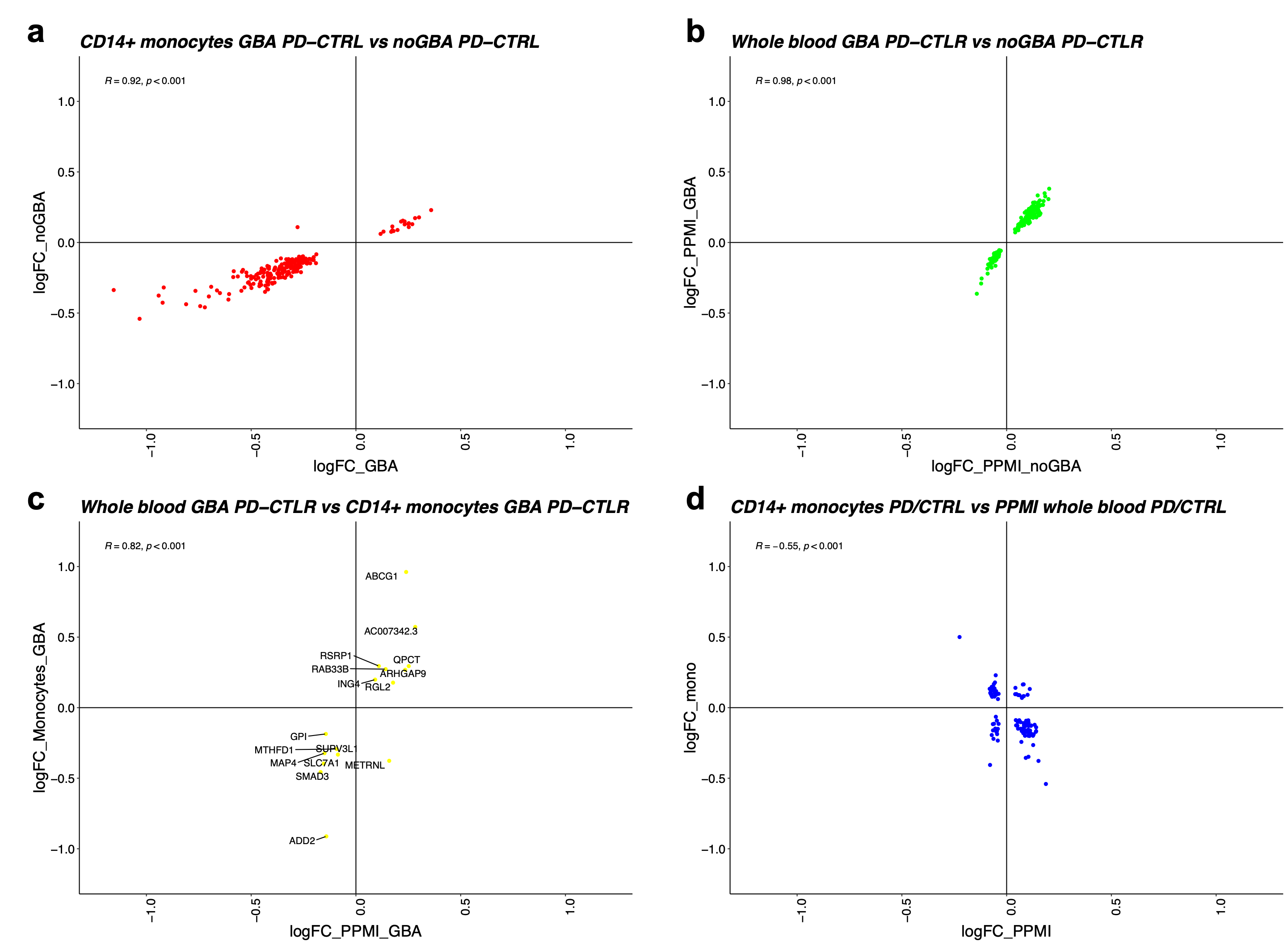


**Supplementary Fig. 10. Correlation between genes differentially expressed in isolated CD14+ monocytes and whole blood.** We compared the directionality of differentially expression genes between a) GBA/PD vs GBA/CTRL and PD vs CTRL in CD14+ monocytes (n = 197); b) GBA/PD vs GBA/CTRL and PD vs CTRL in whole blood (n = 207); c) GBA/PD vs GBA/CTRL in CD14+ monocytes and whole blood (n = 16); d) PD vs CTRL in CD14+ monocytes and whole blood (n = 103).

**Supplementary Fig. 11. Correlation between gene expression levels in isolated CD14+ monocytes and whole blood.**

Genes with expression with more than 1 CPM in 30% of the samples were considered from both cohorts (discovery cohort: isolated CD14+ monocytes (total number of genes: 13711), validation cohort: whole blood - PPMI cohort (total number of genes: 18111)). Spearman correlation between levels of normalized mean gene expression across subjects within each cohort per sub-group of subjects was calculated (R = 0.78  p < 0.001). Genes were normalized with TMM and voom, as detailed in the main text.

**
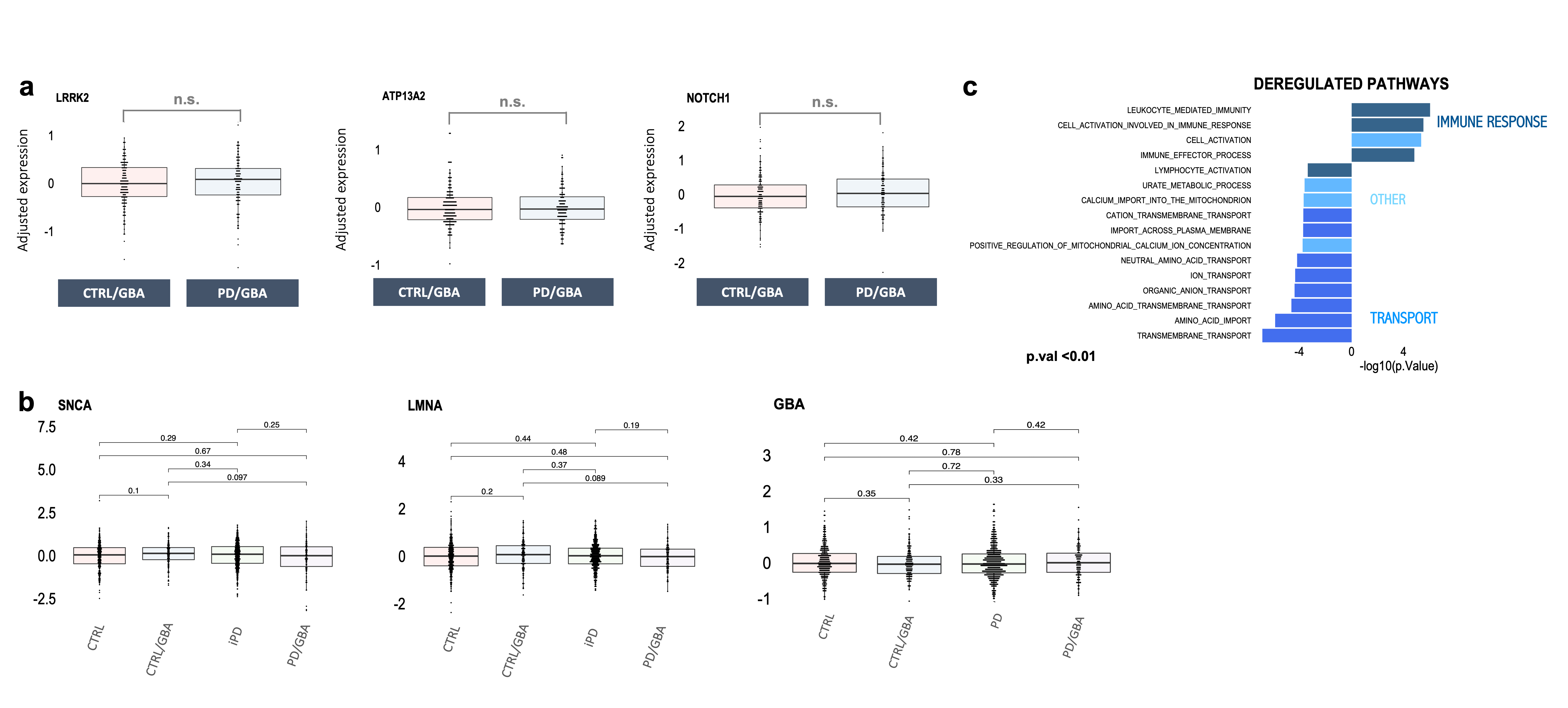
**

**Supplementary Fig. 12. Validation in whole blood of differentially expressed genes in monocytes.**

a) Differential levels of expression of the targeted genes (*ATP13A2*, *LRRK2*, *NOTCH1*, between manifesting and non-manifesting carriers in whole blood from manifesting and non-manifesting GBA-mutation carriers.

b) Differential normalized expression count of *SNCA*, *LMNA,* and *GBA* between PD/GBA and PD, compared to CTRL/GBA and CTRL subjects in whole blood.

In a) and b) each dot represents a subject. Dots are colored based on *GBA* mutations (as reported in the legend: *GBA* mild mutations (N370S, E326K, R496H), *GBA* severe mutations (L444P/A456P/RecNciI, V394L, 84GG, 84GG/T369M, N370S/RecNciI)). p-value of different expression levels is reported on top (statistics: Mann-Whitney U test).

c) Pathway enrichment analysis of differentially expressed genes in whole blood between PD/GBA vs PD subjects with p-value < 0.01 for GO terms are reported. Dark blue: pathways related to cell transport; Green: pathways related to immune response; Light blue: other pathways.
